# Yeast-derived nanoparticles remodel the immunosuppressive microenvironment in tumor and tumor-draining lymph nodes to suppress tumor growth

**DOI:** 10.1038/s41467-021-27750-2

**Published:** 2022-01-10

**Authors:** Jialu Xu, Qingle Ma, Yue Zhang, Ziying Fei, Yifei Sun, Qin Fan, Bo Liu, Jinyu Bai, Yue Yu, Jianhong Chu, Jingrun Chen, Chao Wang

**Affiliations:** 1 Institute of Functional Nano & Soft Materials (FUNSOM), Jiangsu Key Laboratory for Carbon-based Functional Materials and Devices, Soochow University, Suzhou, 215123 Jiangsu China; 2 School of Mathematical Sciences, Soochow University, Suzhou, 215006 Jiangsu China; 3 The Second Affiliated Hospital of Soochow University, Suzhou, 215004 Jiangsu China; 4 National Clinical Research Center for Hematologic Diseases, Jiangsu Institute of Hematology, The First Affiliated Hospital of Soochow University, Institute of Blood and Marrow Transplantation of Soochow University, Suzhou, 215123 Jiangsu China

**Keywords:** Biomedical engineering, Biomaterials, Tumour immunology, Cancer microenvironment

## Abstract

Microbe-based cancer immunotherapy has recently emerged as a hot topic for cancer treatment. However, serious limitations remain including infection associated side-effect and unsatisfactory outcomes in clinic trials. Here, we fabricate different sizes of nano-formulations derived from yeast cell wall (YCW NPs) by differential centrifugation. The induction of anticancer immunity of our formulations appears to inversely correlate with their size due to the ability to accumulate in tumor-draining lymph node (TDLN). Moreover, we use a percolation model to explain their distribution behavior toward TDLN. The abundance and functional orientation of each effector component are significantly improved not only in the microenvironment in tumor but also in the TDLN following small size YCW NPs treatment. In combination with programmed death-ligand 1 (PD-L1) blockade, we demonstrate anticancer efficiency in melanoma-challenged mice. We delineate potential strategy to target immunosuppressive microenvironment by microbe-based nanoparticles and highlight the role of size effect in microbe-based immune therapeutics.

## Abstract (web summary)

Components of the yeast cell wall, including but not limited to β-glucan, have been reported to act as danger signals and promote immune responses. Here the authors report the design and anti-tumor immune responses elicited by yeast cell wall-based nanoparticles in preclinical cancer models.

## Introduction

Cancer immunotherapy is an unprecedented way of utilizing the body’s own immune system to fight tumors^1–3^. However, only a relatively limited (~20%) fraction of patients can benefit from this treatment, including immune checkpoint inhibitor (ICI)-based immunotherapy^4^. A tumor that resists cancer immunotherapy, so-called ‘cold’ tumor^5^, is generally with a poor reflection in cancer cell antigenicity and adjuvanticity^6^, as well as a highly immunosuppressive tumor microenvironment (TME) with limited infiltration by cytotoxic T cells (CTLs) and dendritic cells (DCs) coupled to the accumulation of various myeloid cell populations such as myeloid-derived suppressor cells (MDSCs) and tumor-associated macrophages (TAMs) subsets^7^. The remodeling of immunosuppressive TME has been considered as an important target for the development of combinatorial immunotherapeutic regimens with superior efficiency^8,9^.

Microbe-based cancer immunotherapy has recently emerged as an approach for inducing anticancer immunity. Over the last decade, various microbes, including bacteria^10–13^, oncolytic virus^14,15^ and fungi^16^, have been utilized to activate innate immunity and improve adaptive immunity, thereby augmenting antitumor immune response. For example, it has been reported that engineered attenuated *Salmonella typhimurium* can induce infiltration of immune cells and proinflammatory cytokines production in TME, thereby augmenting antitumor immunity^17^. Several recombinant yeast-based vaccines have been tested in clinical trials^18^. Despite the accumulating data, serious limitations remain^19^. Firstly, due to their potential to cause infections in patients, live microbes may cause the immune system to attack healthy cells, and their use may be in company with the risk of deadly infection. In addition, the facultative anaerobe *Salmonella typhimurium* VNP20009 failed in a phase I clinical trial because the monotherapy was found not enough to eliminate tumor effectively^20^. Microbe-based cancer immunotherapy is still in its early stage ^21^.

Yeast is one of the most common types of fungi, which has been widely used in fermentation and leavening in food manufacture. As the structure of yeast cell wall includes proteins and polysaccharides, such as β-glucan and chitin^22,23^ that do not exist in mammalians, they have been considered as ‘danger signals’, potentially resulting in the activation of potent, multiepitope immune response in our body^24,25^. In addition, yeast-derived β-glucan has been reported to activate DCs and macrophages, thus activating T cells to enhance the anti-tumor efficacy^26^. However, the micro-size of yeast limited their distribution and uptake efficiency in tumor and tumor-draining lymph node. Hence, in this work, we fabricate different sizes of nanoparticles derived from yeast (*Saccharomyces Cerevisiae*) cell wall, which have no reproduction ability. Intriguingly, the induction of anticancer immunity appears to inversely correlate with the size of yeast cell wall nanoparticles (YCW NPs). Small size of YCW NPs (~50 nm) showed better efficiency in controlling tumor growth after intratumor injection compared with middle (~200 nm) and large (~500 nm) size of YCW NPs. By observation of their distribution, we find a high accumulation of small size of YCW NPs in TDLN due to their size effects, as compared with middle and large size of YCW NPs. The mathematic percolation model is introduced to explain their accumulation behavior toward TDLNs. In addition, not only the microenvironment in tumors but also that in the TDLNs are remarkably rebuilt in the abundance and functional orientation of each cellular component following small size YCW NPs treatment. Furthermore, in combination with immune checkpoint blockade therapy, our technology enables complete tumor regression in 90–100% of treated mice with limited side effects. The systematic anticancer immune response is also induced to inhibit the growth of distant tumors. Our study develops microbe-based nanoparticles for enhancing cancer ICI-immunotherapy by remodeling the immunosuppressive microenvironment in tumors and TDLNs, and highlights the role of size effect in microbe-based immune therapeutics.

## Results

### Preparation and characterization of YCW NPs

**Fig. 1:**
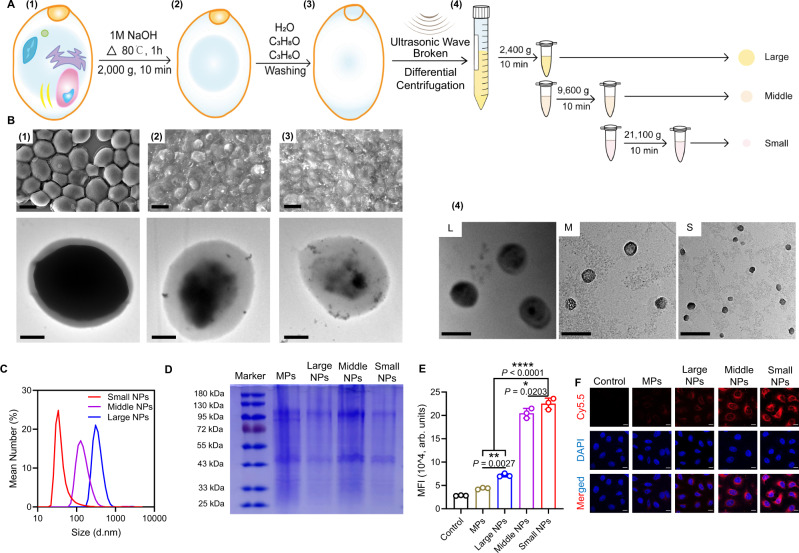
Preparation and characterization of YCW particles. **A** Schematic of preparation of YCW nanoparticles (NPs). **B** SEM and TEM images of every procedure from yeast to different size of YCW NPs. Upper left: SEM image of procedure (1)–(3), Scale bar = 3 μm. Bottom left: TEM image of procedure (1)–(3), Scale bar = 1 μm. Right: TEM image of procedure (4) (three different sizes of YCW NPs), Scale bar = 500 nm. **C** Distribution of three different sizes of YCW NPs observed by DLS. **D** SDS-PAGE of YCW particles, including MPs (micro-particles), Large NPs, Middle NPs and Small NPs, using Coomassie Brilliant Blue Staining. **E** The corresponding quantification of Cy5.5 (MFI) after DC2.4 incubation with Cy5.5-labelled particles for 24 h (*n* = 3, n means the number of samples included in an individual experiment). **F** Confocal imaging of Cy5.5-labelled YCW particles with different sizes after incubation with DC2.4 for 24 h (blue: DAPI; red: Cy5.5; Scale bar = 10 μm, *n* = 3). All experiments were run in triplicate. Statistical significance between different groups was obtained by one-way ANOVA using the Tukey post-test. ****P < 0.0001; **P < 0.01; *P < 0.05. Data are means ± SD. YCW: yeast cell wall, SEM: scanning electron microscopy, TEM: transmission electron microscopy, DLS: dynamic light scattering, MFI: mean fluorescence intensity, arb. units: arbitrary units.

Different nano-size yeast cell walls (YCW NPs) were obtained from the yeast cells. In brief, micro-size yeast cell walls (YCW MPs) were prepared firstly (Fig. 1A). After being broken and washed, yeast cell walls were centrifuged at 2400 *g*, 9600 *g*, and 21,100 *g* for 10 min to obtain three different YCW NPs, named large size of YCW NPs, middle size of YCW NPs, and small size of YCW NPs (Fig. 1A). We measured the compositions of YCW NPs, which contained ~88.20% β-glucan, 2.88% proteins and 8.92% others (Supplementary Fig. 1, Supplementary Table 2, Supplementary Table 3). As shown in scanning electron microscope (SEM) and transmission electron microscope (TEM) imaging, the diameter of YCW MPs were about 4 μm (Fig. 1B*left*), three different sizes of YCW NPs with the diameter of ~50 nm, ~200 nm, and ~500 nm were obtained, respectively (Fig. 1B*right*). Although irregular shapes of NPs were observed (Supplementary Fig. 2A–D), most of them displayed a spherical or quasi-spherical morphology with a uniform of distribution (Fig. 1B*right*, Supplementary Fig. 2A–D). The underlying causes likely include (1) spherical particles are more stable than nonspherical particles because they have lower surface free energy compared to nonspherical particles; (2) particles with irregular shape and surface are more prone to aggregate due to attractive van der Waals interactions during the ultrasonic treatment and removed by centrifugation-based separation procedures. Dynamic light scattering (DLS) showed the similar results as TEM and three kinds of YCW NPs had a similar zeta potential around −12 mV (Fig. 1C, Supplementary Fig. 2E). In addition, sodium dodecyl sulfate-polyacrylamide gel electrophoresis (SDS-PAGE) assay further indicated that all of them had the similar profile of protein contents (Fig. 1D). The changes in diameter and zeta potential at room temperature and 4 °C within 2 weeks were not obvious, indicating great stability of the NPs (Supplementary Fig. 2F–G). We next studied their potential cytotoxicity on DCs (DC2.4), macrophages (RAW264.7), and melanoma (B16-F10) using methyl thiazolyl tetrazolium (MTT) assay, which revealed that three kinds of YCW NPs at indicated concentrations displayed no significant toxicity on both immune cells and tumor cells after incubation for 24 h (Supplementary Fig. 3A–C). These results indicated that the properties of YCW NPs with three different sizes were similar in those aspects.

### YCW particles for activation of immune cells

We then studied the interactions of YCW particles, including YCW MPs and YCW NPs, with DCs and macrophages. Confocal fluorescence imaging and flow cytometry analysis of Cy5.5 indicated that after incubation for 24 h, Cy5.5-labelled YCW particles were taken up by both DCs and macrophages effectively (Fig. 1E,F, Supplementary Fig. 4A–F). It was observed that with increasing sizes, fluorescence intensity of Cy5.5-labelled YCW NPs decreased on DCs, but not obviously on macrophages. It has been reported that β-glucan could be recognized by Dectin-1 expression on DCs and macrophages, thereby activating DCs and macrophages. To confirm the cellular uptake of YCW NPs was related to Dectin-1, we applied Dectin-1 competitor laminarin incubation with immune cells for 2 h before incubation with Cy5.5-labelled YCW NPs. Flow cytometry analysis showed that the recognition and phagocytosis of YCW NPs by DCs and macrophages was, at least in part, related to Dectin-1 (Supplementary Fig. 4G–J).

**Fig. 2:**
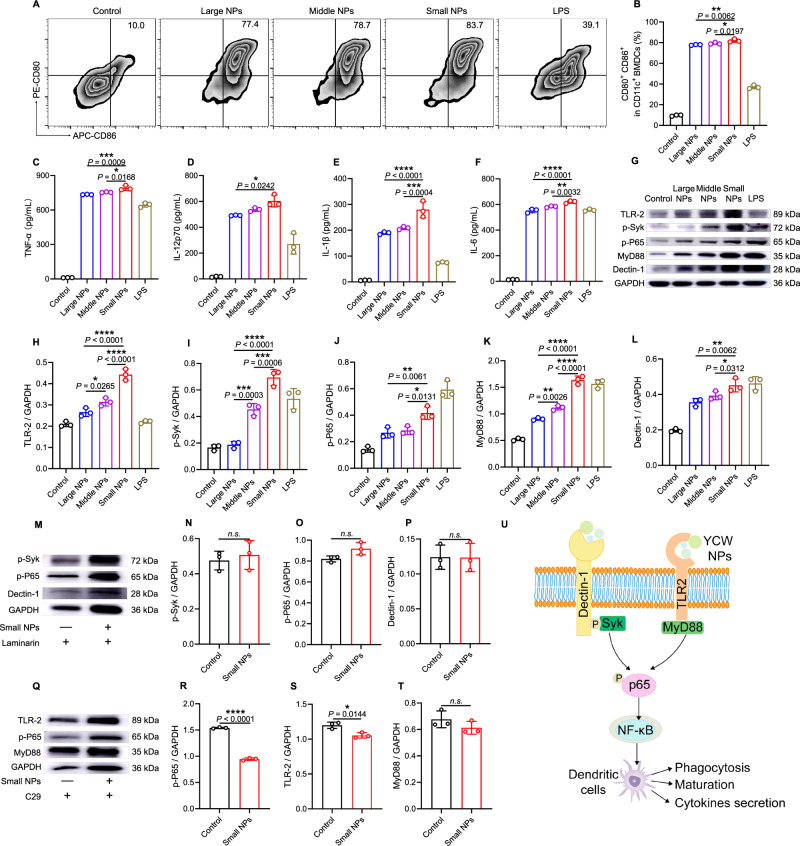
Activation of dendritic cells induced by YCW NPs and its mechanism. **A**–**B** Activation of BMDCs after BMDCs incubation with different sizes YCW NPs (including large NPs, middle NPs, and small NPs) and LPS (positive control) for 24 h. **A** Representative dot plots of co-stimulatory molecules CD80 and CD86 expression on BMDCs and (**B**) corresponding quantification of BMDCs maturation (*n* = 3). **C**–**F** Concentration of pro-inflammatory cytokines secreted by BMDCs after incubation with different sizes YCW NPs as indicated (*n* = 3). **C** TNF-α; **D** IL-12p70; **E** IL-1β; **F** IL-6. **G**–**L** Representative Western blotting result of Dectin-1/Syk pathway and TLR2/MyD88 pathway from proteins of BMDCs after incubation with three YCW NPs and LPS for 24 h (*n* = 3), including (**H**) TLR2, (**I**) p-Syk, (**J**) p-P65, (**K**) MyD88, (**L**) Dectin-1. **M**–**P** After utilizing Dectin-1 competitor laminarin for 2 h, representative western blotting result of Dectin-1/Syk pathway from proteins of BMDCs after incubation with small NPs for 24 h (*n* = 3), including (**N**) p-Syk, (**O**) p-P65, (**P**) Dectin-1. **Q**–**T** After utilizing TLR2 inhibitor C29 for 2 h, representative western blotting result of TLR2/MyD88 pathway from proteins of BMDCs after incubation with small NPs for 24 h (*n* = 3), including (**R**) p-P65, (**S**) TLR2, (**T**) MyD88. **U** A scheme revealing the mechanism of YCW NPs to activate dendritic cells via a Dectin-1 and TLR2-mediated manner. All experiments were run in triplicate. Statistical significance between different groups was obtained by Student’s *t* tests (two-tailed) (**N**–**P**, **R**–**T**) and one-way ANOVA using the Tukey post-test (**B**, **C**–**F**, **H**–**L**). ****P < 0.0001; ***P < 0.005; **P < 0.01; *P < 0.05. Data are means ± SD. BMDCs: bone marrow-derived dendritic cells, LPS: lipopolysaccharides. The samples for western blotting analysis derived from the same experiments and the blots were processed in parallel.

DCs, as a kind of professional antigen-presenting cell, play a vital role in activating a series of adaptive immune responses^27,28^. Therefore, we tested the maturation of bone marrow-derived dendritic cells (BMDCs) treated with three different sizes of YCW NPs. Intriguingly, small size of YCW NPs showed the highest upregulation in co-stimulatory molecules including CD80, CD86, CD40, and MHCII compared with those treated with middle or large size of YCW NPs or MPs (Fig. 2A,B, Supplementary Fig. 5, Supplementary Fig. 6). In line with the result of BMDCs maturation, highest pro-inflammatory factors such as TNF-α, IL-1β, IL-12p70, IL-6 were also produced by the small size of YCW NPs incubation (Fig. 2C–F, Supplementary Fig. 5C–F). These results proved that YCW particles could efficiently activate BMDCs in a size-dependent manner, probably due to their uptake efficiency. At the same time, we also observed the expression of PD-L1 on BMDCs was significantly increased after incubation with YCW NPs (Supplementary Fig. 5I), however, the expression of PD-1, CTLA-4, LAG-3, and TIM-3 did not show such a dramatic change (Supplementary Fig. 5J–M). We next sought to unravel the mechanism whereby YCW NPs activate DCs. Studies have elucidated that yeast cell wall activates DCs and macrophages by Dectin-1/Syk pathway and TLR2/MyD88 pathway^29,30^. We performed western blotting assay to explore whether the YCW NPs activate DCs was also related to these pathways. A dramatic increase was observed in the expression level of TLR2, p-Syk, p-P65, MyD88, Dectin-1 in BMDCs after incubation with three different sizes of YCW NPs. In addition, with the decrease of size, the expression level was increased (Fig. 2G–L, Supplementary Fig. 7A). Next, we applied Dectin-1 competitor laminarin and TLR2 inhibitor C29 to pretreat BMDCs for 2 h. The results future confirmed that activation of BMDCs by YCW NPs was related to, at least in part, Dectin-1/Syk pathway and TLR2/MyD88 pathway (Fig. 2M–U, Supplementary Fig. 7B,C).

T cells play significant roles in immune response. To explore whether YCW NPs directly affect T cells, we incubated T cells with various YCW NPs for 24 h. The results of flow cytometry showed that T cells, unlike DCs and macrophages, could not effectively engulf YCW NPs (Supplementary Fig. 8A,B). At the same time, in vitro experiments revealed that three different sizes of YCW NPs did not promote the activation of T cells and the expression of PD-1 on T cells (Supplementary Fig. 8C–I), suggesting that YCW NPs did not affect T cells directly.

### YCW NPs inhibited tumor growth by remodeling immunosuppressive tumor microenvironment

**Fig. 3:**
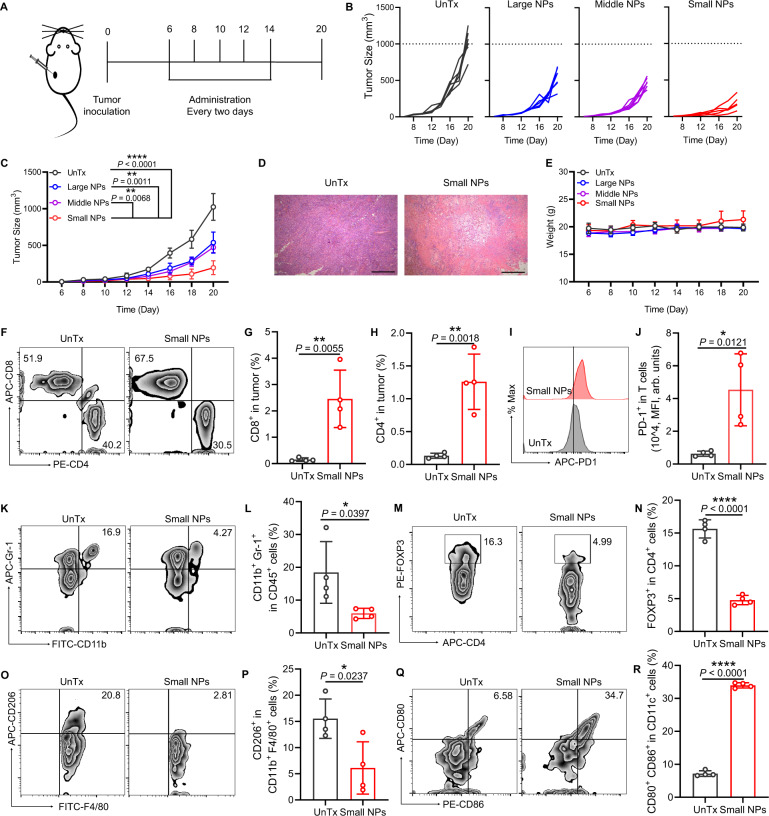
YCW NPs inhibited tumor growth by remodeling immunosuppressive tumor microenvironment. **A** Schematic diagram of therapeutic strategy with YCW NPs, including large NPs, middle NPs and small NPs. **B**–**C** Individual (**B**) and average (**C**) tumor growth curves in groups of untreated and treated with three different sizes of YCW NPs (UnTx: *n* = 6; Large NPs: *n* = 6; Middle NPs: *n* = 6; Small NPs: *n* = 5). **D** Representative H&E staining image of tumors in untreated and small size of YCW NPs treated group (Scale bar = 200 µm, *n* = 3). **E** Weight of mice of four different groups during treatment (UnTx: *n* = 6; Large NPs: *n* = 6; Middle NPs: *n* = 6; Small NPs: *n* = 5). **F** Representative flow cytometric analysis for CD8^+^ and CD4^+^ in tumors and (**G**–**H**) corresponding quantitative analysis (*n* = 4) of untreated group and small NPs group. **I** Representative flow cytometric analysis for PD-1^+^ expression in T cells and (**J**) corresponding quantitative analysis. **K**–**R** Analysis of TME of untreated group and small NPs group. **K** Flow cytometry plots of MDSCs (CD45^+^ CD11b^+^ Gr-1^+^ cells); **L** Proportion of CD11b^+^ Gr-1^+^ in CD45^+^ cells; **M** Flow cytometry plots of Tregs (CD3^+^ CD4^+^ FOXP3^+^ cells); **N** Proportion of FOXP3^+^ in CD4^+^ T cells; **O** Flow cytometry plots of TAMs (CD206^+^ CD11b^+^ F4/80^+^ CD45^+^ cells); **P** Proportion of CD206^+^ in CD11b^+^ F4/80^+^ CD45^+^ cells. **Q** Flow cytometry plots and (**R**) quantification of co-stimulatory factor CD80 and CD86 expression on DCs in tumors. *n* = 4. Statistical significance between different groups was obtained by Student’s *t* tests (two-tailed) (**G**, **H**, **J**, **L**, **N**, **P**, **R**) and one-way ANOVA using the Tukey post-test (**C**). ****P < 0.0001; **P < 0.01; *P < 0.05. Data are means ± SD. MFI: mean fluorescence intensity, arb. units: arbitrary units.

We next questioned whether YCW NPs could inhibit established tumor growth by altering immunosuppressive TME. In our experiment, B16-luc tumor cells were inoculated on the back of C57BL/6 mice and then we intratumorally injected three kinds of YCW NPs (0.375 mg/kg) at a frequency of once every two days for a total of five administrations (Fig. 3A). It was revealed that all YCW NPs inhibited the growth of B16-luc tumor significantly. Intriguingly, among the different size of YCW NPs, the small size of YCW NPs exhibited greatest antitumor efficiency (Fig. 3B,C), which was consistent with our in vitro data. Hematoxylin-eosin staining (H&E staining) of tumor in small size YCW NPs treated group confirmed that tumor cells were eradicated remarkably, which directly proved the remarkable anti-tumor efficacy of small size of YCW NPs (Fig. 3D). The weight of mice remained almost unchanged during the treatment compared to that of untreated control, indicating the limited side-effect of YCW NPs administration (Fig. 3E). Dose–response experiments also identified 0.375 mg/kg YCW NPs effectively inhibited tumor growth (Supplementary Fig. 9). We posited that the antitumor effect of YCW NPs was due to the alteration of immunosuppressive TME. We tested this hypothesis by analyzing the changes in tumor infiltrating immune cells after administration. The total and proportion of tumor infiltrating CD8^+^ T cells and CD4^+^ T cells was significantly increased in small size of YCW NPs treated group (Fig. 3F–H, Supplementary Fig. 10A, B), culminating with an inflamed tumor immune phenotype. However, we also observed the expression of PD-1 on T cells upregulated synchronously by YCW NPs treatment (Fig. 3I–J). Immunosuppressive cellular components including regulatory T cells (Tregs), MDSCs, and TAMs were further analyzed. Flow cytometry data showed that small size of YCW NPs depleted Tregs, MDSCs and TAMs considerably (Fig. 3K–P, Supplementary Fig. 10C–G). In TME, the number and function of DCs are in an impaired state. The presence of MDSCs, Tregs can inhibit the maturation of DCs, resulting in DCs not being able to secret appropriate co-stimulation and cytokine signals to T cells. The maturation of DCs is imperative to provide co-stimulatory signals to T cells, thereby effectively activating naïve T cells. Hence, we assessed the maturation of DCs within tumor. Compared with untreated tumors, the maturity of DCs was as high as about 35% in treated group, which was essential for promoting T cell priming and recruitment (Fig. 3Q–R, Supplementary Fig. 10H–I). Taken together, all these results indicated that immunosuppressive TME was dramatically reversed by YCW NPs treatment (Supplementary Fig. 11).

### Small size of YCW NPs showed great ability to distribute to tumor draining lymph nodes

**Fig. 4:**
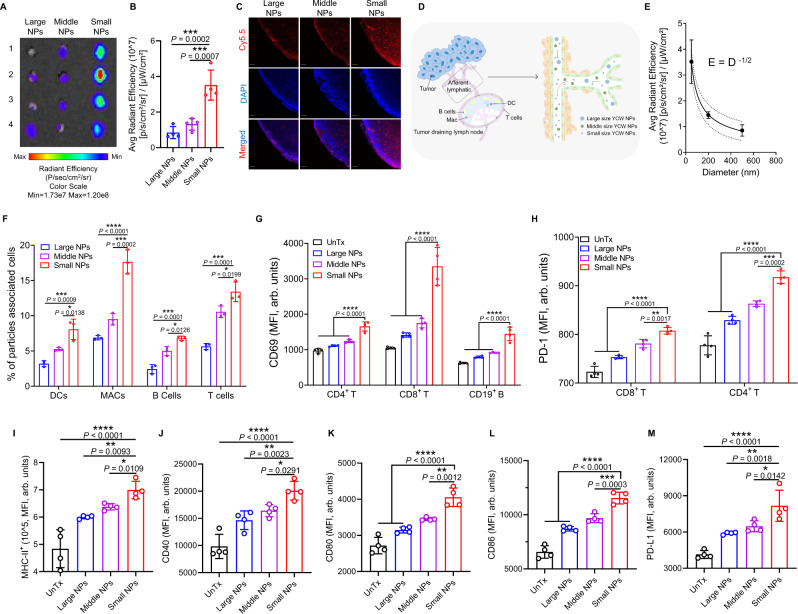
Accumulation of YCW NPs to tumor draining lymph nodes (TDLNs) and activation of immune cells in vivo. **A** Fluorescence imaging of TDLNs ex vivo after injection of three different YCW NPs for 48 h. **B** Signal quantification of Cy5.5-labelled YCW NPs in TDLNs (*n* = 4). **C** Representative confocal imaging of TDLNs to monitor the signal of Cy5.5 (blue: DAPI; red: Cy5.5; Scale bar = 50 µm, *n* = 3). **D** Imaging of draining lymph nodes and model of the distribution of YCW NPs in lymph nodes vessels. **E** Mathematical model to explain the relationship between size and distribution behavior (*n* = 3). **F** Proportions of particles associated cells of tumor draining lymph nodes after intratumorally injection for 48 h (*n* = 3). **G** Corresponding quantification of MFI of CD69 expression on CD4^+^ T cells, CD8^+^ T cells, CD19^+^ B cells (*n* = 4) after injection of YCW NPs for 48 h. **H** Corresponding quantification of MFI of PD-1 expression on CD4^+^ T cells and CD8^+^ T cells (*n* = 4) after injection of YCW NPs for 48 h. **I**–**M** Corresponding quantification of MFI of MHC-II (**I**); CD40 (**J**); CD80 (**K**); CD86 (**L**); PD-L1 (**M**) expression on DCs (*n* = 4) after injection of YCW NPs for 48 h. Statistical significance between different groups was obtained by one-way ANOVA using the Tukey post-test. ****P < 0.0001; ***P < 0.005; **P < 0.01; *P < 0.05. Data are means ± SD. DC: dendritic cells, MFI: mean fluorescence intensity, arb. units: arbitrary units.

We next questioned why small size of YCW NPs showed better efficiency in controlling tumor growth compared to middle and large size of YCW NPs. The innate immune cells within TDLNs are critical for the generation of adaptive responses^31,32^. We reasoned that the distribution efficiency of YCW NPs towards TDLNs might influence their antitumor ability. To validate this hypothesis, three kinds of Cy5.5-labelled YCW NPs were intratumorally injected in B16 tumors. After 48 h, mice were sacrificed and the TDLNs were collected for fluorescence imaging ex vivo. Obviously, we observed that small size of YCW NPs facilitated their entry and targeting in TDLNs, followed by middle size of YCW NPs, while large size of YCW NPs or MPs had poorest ability to target TDLNs (Fig. 4A, Supplementary Fig. 12). Moreover, quantification data revealed that small size of YCW NPs treated mice showed 2.5-fold and 5-fold greater fluorescence in the TDLNs than those treated with the middle size of YCW NPs and large size of YCW NPs, respectively (Fig. 4B). Fluorescent quantitative data further indicated that about 5% of injected small NPs were accumulated in TDLN (Supplementary Fig. 13A,B). Confocal imaging of TDLNs also coincided with the ex vivo imaging (Fig. 4C). We therefore speculated that the distribution efficiency of YCW NPs toward TDLNs was negatively related to the sizes of YCW NPs (Fig. 4D).

Our results were consistent with previous reports. Due to their unique size effect, nanoparticles have a natural tendency to passively draining lymph nodes. For example, studies showed that the size of particles below 200 nm could accumulate to draining lymph nodes fast. However, when the size was above 500 nm, they were limited by extracellular matrix, so that large particles could not get to draining lymph nodes^33,34^. Other research reported that small nanoparticles (10–100 nm) are absorbed by the lymphatic vessels and diffuse to lymph nodes to target DCs. Large nanoparticles (>100 nm) and microparticles (MPs) are mostly embedded in the interstitial matrix, and need to be captured by surrounding immune cells, indicating large nanoparticles are mainly delivered to the lymph nodes in a cell-mediated manner^35^. Data fitting results showed that the distribution capacity of nanoparticles was inversely proportional to the square root of the particle diameter. To explain this phenomenon, the percolation model was introduced. This model is based on stochastic processes, which describes the diffusion of hypothetical liquid particles in a random medium and has been widely used in subsurface hydrology^36^, petroleum engineering, materials science^37^, etc. Lymph nodes are composed of large number of tiny lymphatic vessels connected to each other. A simplified network is used instead of the complex structure of the internal circulation, including blood vessels and lymphatic vessels. When considering a bond percolation problem, the bonds of the network are either occupied (i.e. they are open to flow, diffusion, and reaction) or vacant. The bonds are occupied randomly and independently of each other with probability $p$. One important concept is the bond percolation threshold ${p}_{c}$. Below the threshold, there will be no large clusters (for sites percolation) or long-range connectivity (for bonds percolation). At this point, the nanoparticles would not be able to reach inside the lymph nodes. For nanoparticles of different diameters, the probability of each edge can be estimated by the percolation coefficient $K$. For linear percolation, Darcy’s experimental law shows that $V=-\frac{K{\prime} }{\mu }(\frac{\partial p}{\partial z}+\rho g)$. According to the model, we inferred that the radiation efficiency decreased nonlinearly with the diameter in a certain range. After testing the normal distribution of nanoparticle diameters obtained from each group, the mean value of each group was fitted by logarithmic least squares with respect to the radiation efficiency to obtain the quantitative relationship (Fig. 4E).

To characterize the change of TDLNs immune microenvironment, the distribution of YCW NPs in immune cells of lymph nodes was firstly evaluated. Similar with tumor (Supplementary Fig. 13G), signals of Cy5.5 were detected in main TDLNs immune cells (Fig. 4F*and* Supplementary Fig. 13C–F), including DCs, macrophages, B cells and T cells, indicating that YCW NPs were distributed in immune cells of TDLNs and its contents appeared to inversely correlate with size of YCW NPs. The small size of YCW NPs had the stronger capacity to entry into these immune cells compared to middle and large ones. In contrast, non-TDLNs did not show any significant Cy5.5 intensity (Supplementary Fig. 13H). In view of distribution of YCW NPs in tumor infiltrating immune cells (Supplementary Fig. 13G), the transport of YCW NPs to lymph nodes was not only confined to simple diffusion, but also can be mediated by local immune cells in the tumor. Next, we explored the ability of three sizes of YCW NPs to regulate the microenvironment of TDLNs. Both T (CD4^+^ and CD8^+^) cells and B (CD19^+^) cells upregulated the expression of CD69 after 48 h of injection, indicating that both T cells and B cells were activated by the treatment (Fig. 4G). PD-1 is also expressed during the early phase of T cell activation^38^. Consistent with the results of tumor analysis, the expression of PD-1 on T cells upregulated synchronously by YCW NPs treatment, indicating YCW NPs could effectively activate T cells in both tumor and TDLNs (Fig. 4H). Next, we analyzed the expression of co-stimulatory molecules on DCs of TDLNs. The results showed that co-stimulatory molecules, such as MHCII, CD40, CD80, and CD86 were all increased in the treatment group compared with those in control group (Fig. 4I–L). Meanwhile, the expression of PD-L1 on DCs reflected the same trend (Fig. 4M), which might limit T cell responses. Together, these results suggested that compared with middle and large size of YCW NPs, small size of YCW NPs induced higher stimulatory markers on various immune cells in the mice, mainly due to their highest accumulation in TDLNs.

### T-cell-mediated anti-tumor immune responses induced by small size of YCW NPs

**Fig. 5:**
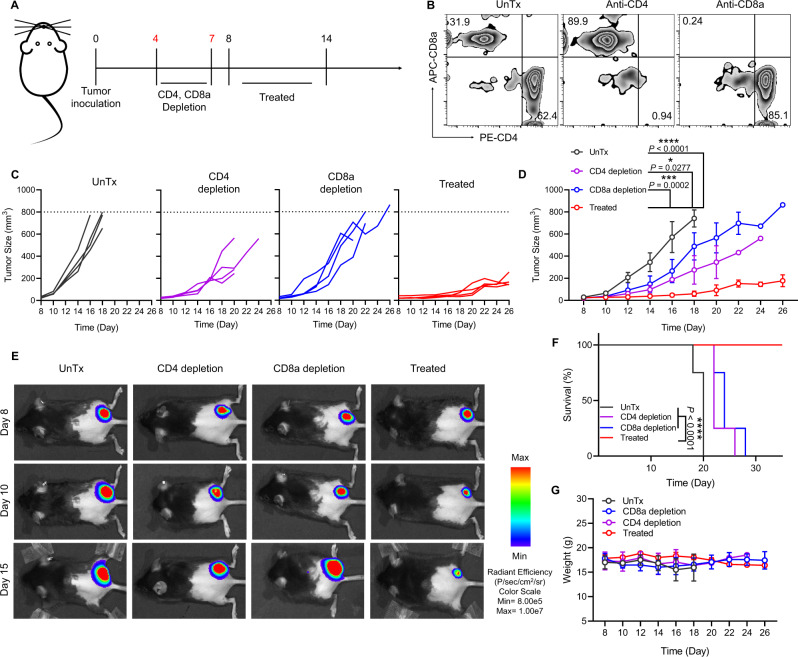
T-cell-mediated anti-tumor immune responses induced by small size of YCW NPs. **A** Schematic diagram of therapeutic strategy with T cell depletion, including CD4 depletion and CD8a depletion. **B** Representative flow cytometric analysis for CD8^+^ and CD4^+^ in CD3^+^ T cells in peripheral blood to confirm the depletion of CD4 and CD8a in vivo. **C**–**D** Individual (**C**) and average (**D**) tumor growth curves in four groups, including UnTx group, CD4 depletion group, CD8a depletion group and treated group. **E** Fluorescence imaging of B16-luc established mice on day 8, 10, 15 in four different groups. **F** Survival curves for the depletion groups and treated group. **G** Weight curves of different groups. Statistical significance was obtained by one-way ANOVA using the Tukey post-test (*n* = 4). ****P < 0.0001; ***P < 0.005; *P < 0.05. Data are means ± SD. MFI: mean fluorescence intensity, arb. units: arbitrary units.

As the YCW NPs encompassed ‘danger signals’ which may result in the activation of various innate immune cells, we next applied anti-CD4 antibody and anti-CD8a antibody to deplete T cells in vivo to see if the anticancer immune responses caused by YCW NPs were related to T-cell-mediated adaptive immunity. Before treatment, we performed T cell depletion by applying the antibodies on day 4 and day 7, respectively (Fig. 5A). Flow cytometry analysis of ratio of CD4^+^ T cells and CD8^+^ T cells in the serum was carried out to determine whether the corresponding T cells were completely depleted (Fig. 5B, Supplementary Fig. 14). After depletion of T cells successfully, we started administrating small NPs. The growth of tumor in both CD4^+^ and CD8^+^ T cell depletion groups increased faster than treated group (Fig. 5C–E), leading to diminished immunotherapy efficiency as determined by survival analysis (Fig. 5F–G). These data indicated that T-cell-mediated anti-tumor immune responses was indispensable for YCW NPs-based cancer immunotherapy.

### Therapeutic efficacy of YCW NPs in combination with PD-L1 blockade

**Fig. 6:**
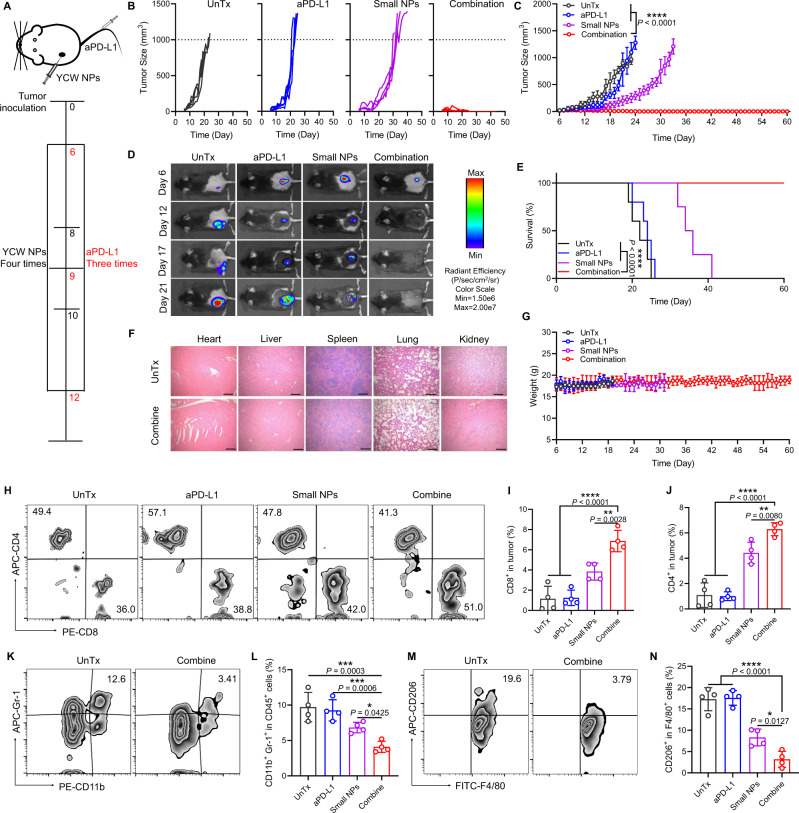
Therapeutic efficacy of YCW NPs in combination with PD-L1 blockade. **A** Schematic diagram of combination treatment, including UnTx group, aPD-L1 group, small NPs group, combination group. **B**–**C** Individual (**B**) and average (**C**) tumor growth curves in four groups (UnTx: *n* = 5; aPD-L1: *n* = 5; Small NPs: *n* = 4; Combination: *n* = 6) within 60 days. **D** In vivo bioluminescence imaging of B16-luc established mice in four different groups. **E** Survival curves of combination therapy. **F** Representative H&E staining imaging of main organs, including heart, liver, spleen, lung, kidney in group of untreated and combination (Scale bar = 200 µm, *n* = 3). **G** Weight curve of different groups. **H** Flow cytometry plots of CD4^+^ T cells and CD8^+^ T cells of tumors in four groups. **I** Corresponding quantitative analysis of CD8^+^ T cells of tumor. **J** Corresponding quantitative analysis of CD4^+^ T cells of tumor. **K** Flow cytometry plots of MDSCs (CD45^+^ CD11b^+^ Gr-1^+^ cells) in untreated and combination group. **L** Corresponding quantitative analysis of CD11b^+^ Gr-1^+^ in CD45^+^ cells. **M** Flow cytometry plots of TAMs (CD206^+^ CD11b^+^ F4/80^+^ CD45^+^ cells) in untreated and combination group; **N** Corresponding quantitative analysis of CD206^+^ in CD11b^+^ F4/80^+^ CD45^+^ cells (*n* = 4). Statistical significance between combination group and other groups was obtained by one-way ANOVA using the Tukey post-test. ****P < 0.0001; ***P < 0.005; **P < 0.01. Data are means ± SD.

In our previous data, we found that both PD-1 on T cells and PD-L1 on DCs were significantly upregulated following the YCW NPs treatment (Fig. 3J, Fig. 4H,M). We speculated that blockade of PD-1/PD-L1 by antibody in combination with small size of YCW NPs might induce synergetic antitumor effect. To test this, we established a B16-luc melanoma model in C57BL/6 mice. Small size YCW NPs was administrated intratumorally at a frequency of once every two days for a total of four times. Anti-PD-L1 mAbs were injected intravenously for three times at three-day intervals (Fig. 6A). As expectedly, YCW NPs based immunotherapy showed great synergy with PD-L1 blockade in controlling tumor growth as compared with monotherapy (Fig. 6B–D). More encouraging, the combination treatment enabled complete tumor regression in 100% of treated mice (Fig. 6B–D), which was translated into significantly lengthened survival (Fig. 6E). Noteworthily, the combination treatment was well tolerated in the mice as evidenced by the slight weight change of mice and H&E staining of main organs (Fig. 6F–G, Supplementary Fig. 15A). By analysis of various immune components in tumor, we found that the combination treatment was sufficient to augment the infiltration of CD8^+^ T cells and CD4^+^ T cells into the tumors, indicating a robust T-cell mediated anti-tumor immunity (Fig. 6H–J). In addition, the frequency of MDSCs and TAMs in TME were remarkably reduced following the combination treatment (Fig. 6K–N, Supplementary Fig. 15B–C). All these data suggested that small size YCW NPs significantly synergized with PD-L1 blockade in inducing a robust antitumor immunity.

### YCW NPs in combination with PD-L1 blockade inhibited metastatic tumor growth

**Fig. 7:**
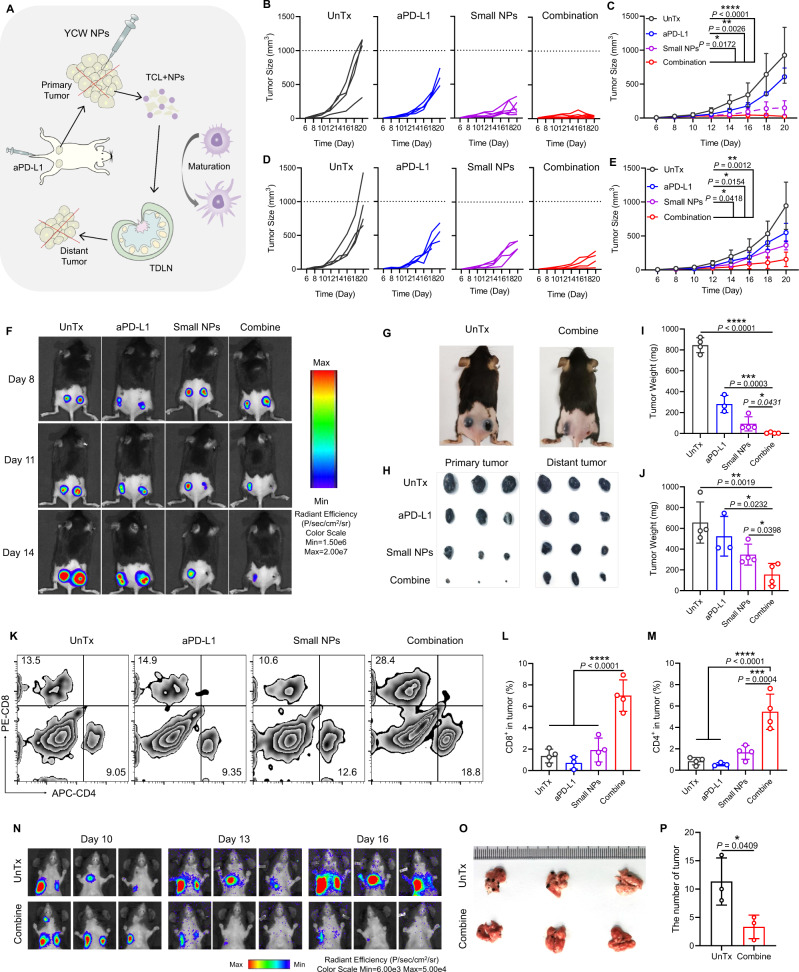
YCW NPs in combination with PD-L1 blockade inhibit metastatic tumor growth. **A** Schematic diagram of applying combination therapy to induce anti-tumor immune responses systemically. **B**–**C** Individual (**B**) and average (**C**) tumor growth curves in four groups of primary tumors, including UnTx group (*n* = 4), aPD-L1 group (*n* = 4), small NPs group (*n* = 6), combination group (*n* = 6). **D**–**E** Individual (**D**) and average (**E**) tumor growth curves of distant tumors (*n* = 4) in four different groups. **F** Fluorescence imaging of B16-luc established mice on day 8, 11, 14. **G** Representative photographs of mice in untreated and combination group on day 14. **H** Photographs and **I**–**J** weight of primary tumors and distant tumors within 20 days (UnTx: *n* = 4; aPD-L1: *n* = 3; Small NPs: *n* = 4; Combine: *n* = 4). **K** Flow cytometry plots of CD4^+^ T cells and CD8^+^ T cells of distant tumors in four groups. **L** Corresponding quantitative analysis of CD8^+^ T cells of distant tumor. **M** Corresponding quantitative analysis of CD4^+^ T cells of distant tumor (*n* = 4). (**N**) Bioluminescence imaging of lung metastasis established by B16-luc in vivo on day 10, 13, 16 in untreated and combination groups. **O** Imaging of whole lung tissue ex vivo. (**P**) Number of tumors in lung (*n* = 3). Statistical significance between different groups was obtained by Student’s *t* tests (two-tailed) **P** and one-way ANOVA using the Tukey post-test (**C**, **E**, **I**, **J**, **L**, **M**). ****P < 0.0001; ***P < 0.005; **P < 0.01; *P < 0.05. Data are means ± SD.

The combination of small size of YCW NPs with anti-PD-L1 significantly inhibited the growth of B16-luc locally by destroying tumor to generate tumor cell lysates (TCLs). TCLs and small size of YCW NPs were jointly delivered to TDLNs to promote the maturation of DCs and activation of T cells and B cells (Fig. 7A). Next, we wondered whether local immunotherapy could be sufficient to induce systemic immune response to dampen the growth of distant tumors. Therefore, on day 0, we inoculated B16-luc tumor cells on both sides of the back of C57BL/6 mice. When the tumors grew up to 50 mm^3^, the same approaches and dosage were applied. It was observed from the growth curve that by local therapy, not only the treated tumors, but also the tumors on the opposite side regressed remarkably (Fig. 7B–G, Supplementary Fig. 16). After mice were sacrificed, the tumors were collected and weighed. It was showed that the weights of tumors on both sides of the combination treatment group were lower than that of the other treatment groups and the control group (Fig. 7H–J), indicating that the combination treatment induced systemic anti-tumor immune responses. Flow cytometry analysis reflected that the frequency of CD8^+^ T cells and CD4^+^ T cells in distant tumors increased after combination treatment (Fig. 7K–M). We further explored whether injected small size YCW NPs locally could treat lung metastasis of melanoma. Encouragingly, we found that the lung metastatic tumor growth was also regressed as indicated by the bioluminescence imaging (Fig. 7N) and reduced the number of metastatic tumor foci on lungs after necropsy (Fig. 7O–P). The results intuitively indicated that local administration of small size YCW NPs combined with PD-L1 blockade could dramatically produce systemic anti-tumor immune responses to promote metastatic tumor regression.

**Fig. 8:**
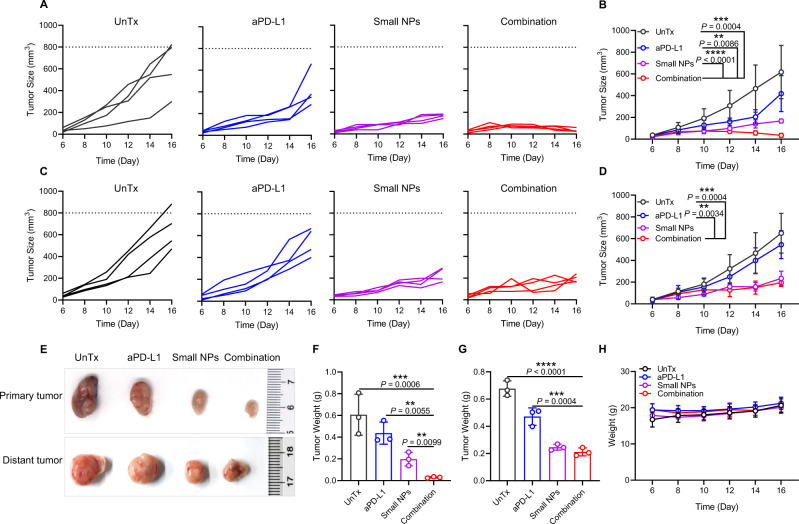
Combination treatment induced systemic anti-tumor immune response against CT26 tumor. **A**–**B** Individual (**A**) and average (**B**) tumor growth curves in untreated and treated groups of primary tumors, including UnTx group, aPD-L1 group, Small NPs group, combination group. **C**–**D** Individual (**C**) and average (**D**) tumor growth curves of distant tumors in four different groups (*n* = 4). **E** Photographs and (**F**–**G**) corresponding quantitative weight of primary tumors and distant tumors of four groups (*n* = 3). **H** Weight of mice in four groups during treatment (*n* = 4). Statistical significance was obtained by one-way ANOVA using the Tukey post-test. ****P < 0.0001; ***P < 0.005; **P < 0.01. Data are means ± SD.

To further demonstrate that our proposed YCW NPs-based immunotherapy could be broadly applicable, we subsequently constructed tumor model of CT26 on BALB/c mice. On day 0, we injected CT26 tumor cells on both sides of back of BALB/c mice. On the sixth day, we administrated small size YCW NPs at one tumor locally, and injected the anti-PD-L1 antibody intravenously. The frequency and dosage of drugs was same as before-mentioned. We again observed a sharp inhibition in tumor growth in the CT26 model. Tumor growth curves on primary tumors and distant tumors suggested that tumors of both sides were remarkably suppressed (Fig. 8A–D). On day 17, we collected tumors for weighing, and result was agreement with that shown in curves of tumor growth (Fig. 8E–G). During the process of therapy, the weight of mice remained almost unchanged (Fig. 8H), indicating that our therapy approach did not produce obvious side effects in the mice.

## Discussion

In recent years, microbe-based cancer therapy has attracted much attention due to its rapid progress. Nevertheless, it still has not achieved satisfactory effects in clinic. It has been documented that bacterial therapy merely resulted in a low tumor regression rate but produced undesirable dose-dependent side effects^11,39^. Oncolytic viruses can induce potent immune responses through generating antitumor cytokines. However, they can also cause serious systemic toxicity^40–42^. Fungi, as one kind of eukaryotic microbe, own similar structure with mammals. Various fungi have been utilized as food, and for use of food manufacture in the long history of humans. Yeast is one of the most common types of fungi with a relatively good biosafety. In addition, yeast-derived β-glucan has been considered as immuno-stimulating agent to treat cancer ^43^.

To address the challenges of microbe-based cancer therapy, in this work, we prepared nanoparticles derived from yeast cell wall. Compared with live yeast cells, the YCW NPs have no activity of reproduction, as well as a much smaller size than yeast cells. To study whether the size effect influences the therapeutic efficiency, three different sizes of YCW NPs were prepared by differential centrifugation. They had no obvious differences in loading of proteins and polysaccharides. Different from macrophages, DCs showed cellular uptake of YCW NPs in a size-dependent manner in the range of 50–500 nm. Previous studies showed that smaller nanoparticles are more easily uptake into the DCs. For example, compared with PLGA nanoparticles with a diameter of more than 1 μm, PLGA NPs with a diameter of 300 nm have a higher DCs uptake efficiency^44,45^. Perhaps due to the greater ability of macrophages to engulf and digest foreign particles^46^, NPs uptake by macrophages is not affected by the particle size significantly in the range from 50 to 500 nm. Nevertheless, we also observed that macrophages showed greater ability to phagocytosis YCW NPs than YCW MPs.

The tumor and TDLNs immune microenvironment regulation are mediated by components of YCW NPs, such as β-glucan which is immunomodulatory compound to activate DCs and macrophages within tumor and TDLNs^47^. Intriguingly, we found significantly-improved tumor regression following intratumorally injection of YCW NPs. Furthermore, we found that this anticancer effect caused by YCW NPs was inversely correlated with their sizes, that was, with the decrease of diameters, tumors were suppressed more efficiently. The innate immune cells within TDLNs is critical for the generation of adaptive responses^31^. Therefore, we were interested to explore whether the small size of YCW NPs could promote their TDLNs accumulation. As expected, we observed that small size of YCW NPs facilitated their entry and targeting in TDLNs, followed by middle size YCW NPs, while large size of YCW NPs had the poorest ability to target TDLNs. The alteration of TDLNs immune microenvironment was also evaluated. As a result, the abundance and functional orientation of each effector subset by small size of YCW NPs treatment were significantly improved not only in the microenvironment in tumors but also in the TDLN, the latter of which might be the key site for initiation of greatest anticancer efficiency in response to small size of YCW NPs. As a natural nanoparticle, all composition of which can together to trigger antitumor immune responses. For example, yeast-derived β-glucan has been considered as a major immuno-stimulating agent to treat cancer^26^. Other yeast-derived components including protein, lipid, and nucleic acid^48,49^ may also play roles in remodeling immunosuppressive TME, which should be investigated in the future work. Glucan particles (GPs) which have already been described for a variety of applications by the Levitz group and others^50–52^ can serve as an effective adjuvant and vaccine platform for cancer immunotherapy. Our nano-formulate yeast cell wall (YCW NPs) is produced from “GPs” (Fig. 1A-B). However, the GPs showed a diameter with ~4 μm, limiting their ability of entry and targeting in TDLNs (Supplementary Fig. 12) as well as therapeutic outcomes (Supplementary Fig. 17) compared to YCW NPs (~50 nm). We indicate the role of particle size in inducing anticancer immunity.

We then used the mathematic percolation model to explain this phenomenon. It visually demonstrated the influence of size effects with mathematical model. To our knowledge, this is the first study of size-dependent immunotherapy based on nanoparticles derived from yeast, delineating theoretical basis for how the size effect influences the efficiency of cancer immunotherapy. Although in this work we mainly studied the role of size effect of YCW NPs, the role of other properties, such as charge densities, in controlling tumor growth and activating immune cells is an issue that warrants further investigation.

Given that both PD-1 on T cells and PD-L1 on DCs were significantly upregulated following the YCW NPs treatment (Fig. 3J, Fig. 4H, M), we speculated that blockade of PD-1/PD-L1 in combination with small size YCW NPs was capable to induce synergetic antitumor effect. To maximize the therapeutic outcomes, PD-L1 blockade therapy was then introduced to synergy with YCW NPs. Strikingly, the combination treatment enabled complete tumor regression in 100% of treated mice, significantly lengthened survival. Moreover, the systematic anticancer immune response was also triggered to restrain the growth of distant tumor following combination therapy. Importantly, we found the mice could well tolerate the combination treatment through monitoring the change in body weight together with histological examination of main organs, suggesting that the combination treatment did not elicit apparent cytotoxicity and thus appeared safe for the mice.

In conclusion, we developed a yeast-based nanoparticle for enhancing cancer immunotherapy. We also studied how size effect of NPs influenced the anticancer efficiency. The small size of YCW NPs dramatically inhibited tumor growth by remodeling the immunosuppressive microenvironment in both the tumors and TDLN. In combination with PD-L1 blockade, we demonstrated an excellent anticancer efficiency in treating melanoma-bearing mice. We delineated potential strategy to target immunosuppressive microenvironment with microbe-based nanoparticles and highlighted the role of size effect in microbe-based immune therapeutics. Nanoparticles comprised of polysaccharides also act as danger signals to provoke immune response^53,54^. However, most of them are synthesized nanoparticles and need complicated chemical reactions with high level of skills and cost. In contrast, the preparation of nano-formulate YCW is very simple, including ultrasonic treatment and centrifugation-based separation procedures. In addition, the size of YCW is tunable by differential centrifugation. Due to its ease of preparation, low-cost, feasibility and biosafety of yeast-derived nanoparticles, this technology may have promising clinical translation potential.

## Methods

This research complies with all relevant ethical regulations approved by Soochow University.

### Materials

Yeast and antibodies applied in this study were shown in Supplementary Table 1.

### Cell lines

Melanoma B16-luc (catalog number: fh1123), CT26 (catalog number: TCM37), RAW264.7 (catalog number: TCM13), and DC2.4 (catalog number: fh1026) were purchased from Cell Bank, Shanghai Institutes for Biological Sciences, Chinese Academy of Sciences. B16-luc cells and RAW264.7 cells were maintained in Dulbecco’s modified Eagle’s medium (DMEM) containing 10% fetal bovine serum (Gibco), penicillin (100 U/ml; Invitrogen), and streptomycin (100 U/ml; Invitrogen). CT26 cells and DC2.4 cells were maintained in RPMI 1640 medium containing 10% fetal bovine serum (Gibco), penicillin (100 U/ml; Invitrogen), and streptomycin (100 U/ml; Invitrogen). BMDCs were isolated from bone marrow cavities of 7–8-week-old C57BL/6 mice according to an established approach^55,56^. In brief, bone marrow cells were isolated from female C57BL/6 mice, then incubated in RPMI 1640 medium with GM-CSF (20 ng/mL) for seven days. At last, bone marrow DCs were utilized at 7 days.

### Mice

C57BL/6 and BALB/c female mice (6–8 weeks) were obtained from Nanjing Peng Sheng Biological Technology Co. Ltd. We performed all mice studies in accordance with the animal protocol approved by our university laboratory animal center (Approval number: SUDA20210201A02). Experimental group sizes were approved by the regulatory authorities for animal welfare after being defined to balance statistical power, feasibility and ethical aspects. Maximal tumor burden permitted is 1200 mm^3^. In some cases, this limit has been exceeded the last day of measurement and the mice were immediately euthanized. The actual tumor size (even if larger than 1200 mm^3^) has been recorded and presented in the Article and Source data file.

### Preparation and characterization of nanoparticles with different sizes

To form nanoparticles, the yeast cells were incubated in NaOH at 80 °C for 1 h. After cooled down to room temperature, the lysis of yeast cells was centrifuged at 2000 *g* for 10 min to obtain insoluble matter containing cell walls. Then it was washed with H_2_O, isopropanol, and acetone, and centrifuged to obtain micro-sized cell walls. After broken by ultrasonic cell disruptor, NPs with different sizes were obtained by differential centrifugation. Specifically, 25 mg micro-sized cell walls were dissolved in PBS (pH = 7.4), and after crushing, precipitate obtained at 2400 *g* for 10 min was resuspended in PBS to form large size of YCW NPs. Insoluble matter obtained by centrifuging supernatant at 9600 *g* for 10 min was middle size of YCW NPs. Next, supernatant centrifuged at 21,100 *g* for 10 min to form small size of YCW NPs. The morphologies of yeast cell wall (YCW MPs) were observed by scanning electron microscopy (SEM) and transmission electron microscopy (TEM). Three YCW NPs were characterized by TEM. In detail, we activated the carbon-coated 400-mesh grid firstly, then dropped YCW particles on the grids, and washed with H_2_O. Finally, we observed YCW particles by TEM after dried. We utilized double sticky carbon tape to deposit the silicon wafer dripped with YCW particles on the sample stage, then dried it overnight for SEM. Size distribution and zeta potential of three YCW NPs in aqueous solution were observed by Zetasizer nano ZS instrument. The stability of different YCW NPs was monitored at 4 °C and room temperature in 2 weeks. SDS-PAGE and BCA assay were utilized to evaluate the expression and concentration of protein of different YCW NPs. To determine the composition of polysaccharides, the polysaccharides were first hydrolyzed into monosaccharides. Then the kinds and proportions of monosaccharides were determined by high performance liquid chromatography. We converted proteins into peptides with enzyme digestion assay, and then utilized LC-MS/MS to determine proteins contained in YCW NPs by searching through the protein database. The composition of YCW NPs was determined and analyzed by Shanghai Fuda Analytical Testing Group.

### In vitro cytotoxicity assessment

The cytotoxicity of three YCW NPs to DCs (DC2.4), macrophages (RAW264.7) and B16 was assessed by MTT assay following the standard protocol. In brief, the cells were seeded into 96-well plate at 10^5^ cells/mL for 12 h, and then incubated with three YCW NPs for 24 h. The concentrations of YCW NPs incubated with DC2.4 and RAW264.7 were from 0 μg/mL to 150 μg/mL. The concentration of YCW NPs to B16 was 150 μg/mL. Subsequently, MTT solution was added to each well and the plates were incubated for 4 h. At the end of incubation, supernatants were aspirated, and 100 µL of dimethyl sulfoxide was added to each well with shaking for 10 min at dark to dissolve formazan crystals. The absorbance was measured at 570 nm by a microplate reader.

### In vitro cellular uptake and BMDCs maturation assay

To evaluate the ability of cellular uptake of YCW particles, including MPs, large NPs, middle NPs, and small NPs, DC2.4, BMDCs and RAW264.7 were incubated with Cy5.5, or Cy5.5-labelled YCW particles for 24 h. The amount of Cy5.5-labelled YCW particles phagocytosed by antigen-presenting cells (APCs) was judged by flow cytometry and confocal microscopy analysis. In experiment of flow cytometry, DC2.4 and BMDCs were first stained with FITC-CD11c, then the mean fluorescence intensity (MFI) of Cy5.5 on FITC-CD11c^+^ cells was measured. Similarly, after staining RAW264.7 with FITC-F4/80, MFI of Cy5.5 was also measured. In confocal assay, DC2.4, BMDCs and RAW264.7 were incubated with different preparations, including Cy5.5 and Cy5.5-labelled YCW particles, for 24 h. After washing with PBS for three times, cells were fixed with 4% paraformaldehyde for 30 min. Next, cell nuclei were stained with DAPI (4′,6-diamidino-2-phenylindole) for 10 min. The phagocytic ability of APCs (such as DCs and macrophages) was observed by confocal microscope (Zeiss LSM 800). In order to verify whether cellular uptake of YCW NPs by DCs and macrophages was related to Dectin-1, we applied Dectin-1 competitor laminarin (100 μM) to pretreat DCs and macrophages for 2 h. Then DCs and macrophages were incubated with YCW NPs for 24 h. The results of cellular uptake were analyzed by flow cytometry and confocal assay as before. To explore the effect of YCW NPs, including large NPs, middle NPs, and small NPs, on T cells, we incubated cells obtained from inguinal lymph nodes with YCW NPs for 24 h. Then we analyzed the influences of YCW NPs on T cells. Specifically, after cells were incubated with Cy5.5-labelled YCW NPs for 24 h, the MFI of Cy5.5 was analyzed by flow cytometry to determine the phagocytosis of YCW NPs by T cells. After cells obtained from inguinal lymph nodes were incubated with YCW NPs for 24 h, the activation of T cells was detected by flow cytometry, including the expression of CD69 and PD-1 on CD4 T cells and CD8 T cells.

In the assay of maturation of BMDCs, 5 × 10^5^ immature BMDCs were seeded on 24-well plates, after incubated with PBS, lipopolysaccharides (LPS, positive control), YCW particles, including MPs, large NPs, middle NPs, and small NPs, for 24 h, BMDCs were collected at 100 g for 3 min, and supernatants were harvested for enzyme-linked immunosorbent assay (ELISA). The collected BMDCs were resuspended in FACS buffer (PBS containing 1% FBS) and stained with FITC-CD11c, APC-CD86, PE-CD80, PE-MHCII, APC-CD40, PE-PD-L1, APC-PD-1, PE-CTLA-4, PE-LAG-3, PE-TIM-3 at room temperature for 30 min before analyzing by flow cytometry (BD Accurit C6 Plus). The concentration of inflammatory factors secreted in supernatants, such as TNF-a, IL-1β, IL-12p70, IL-6, was measured by ELISA (BioLegend) according to the manufacturer’s instructions.

To explore the mechanism of activation of DCs by YCW NPs, we carried out western blotting assay. The total proteins of BMDCs pre-incubated with three different sizes of YCW NPs for 24 h were extracted, and the expression of TLR-2, p-Syk, p-P65, MyD88, Dectin-1 was detected by western blotting. In brief, cells were lysed, using RIPA lysis buffer with PMSF protease inhibitor and phosphatase inhibitor obtain total proteins, which were then separated the protein by 12.5% SDS-PAGE gel electrophoresis. Next, proteins were transferred to PVDF membrane by wet method. The membrane was blocked with 3% BSA solution, and incubated with anti-p-P65, anti-TLR-2, anti-p-Syk, anti-MyD88, anti-Dectin-1 and anti-GAPDH antibody overnight, and then incubated with rabbit secondary antibody (Goat anti-Rabbit IgG (H+L)-HRP) for 1 h. The bands were developed by chemiluminescence, and the image was quantitatively analyzed with Image J software. To further confirm the activation of DCs via Dectin-1/Syk pathway and TLR2/MyD88 pathway, we pretreated BMDCs with Dectin-1 competitor laminarin (100 μM) and TLR2 inhibitor C29 (50 μM) for 2 h, respectively. After incubated with small NPs for 24 h, proteins of BMDCs were collected for western blotting assay as before.

### Analysis of lymph nodes ex vivo

To analyze the accumulation of YCW particles, including MPs, large NPs, middle NPs and small NPs toward tumor draining lymph nodes (TDLNs), C57BL/6 mice were intratumorally injected with Cy5.5-labelled YCW particles. After 48 h, mice were sacrificed and TDLNs were harvested for imaging by IVIS Spectrum Imaging System and confocal microscopy. At the same time, tumors, TDLNs and non-TDLNs were collected for flow cytometry. The tumors, TDLNs and non-TDLNs were grinded and digested, and then stained with FITC-CD11c, FITC-F4/80, FITC-CD19, FITC-CD3 antibodies to evaluate the number of different NPs in DCs, macrophages, B cells and T cells. Meanwhile, to observe the distribution of YCW particles toward TDLNs more intuitively, TDLNs from different groups were buried in optimal cutting temperature compound, then cut into micrometer slices and fixed on slides. After staining cell nucleus with DAPI, the sections were observed using confocal microscope. In order to analyze the activation status of immune cells in TDLNs, PE-MHCII, APC-CD40, PE–CD80, APC–CD86 were stained to observe the maturation of DCs. APC–CD69 staining was used to evaluate the activation state of T cells and B cells. At the same time, APC–PD-1 and APC–PD-L1 staining was applied to examine the expression levels of PD-1 and PD-L1 on T cells and APCs, respectively.

### Therapeutic experiments of YCW NPs

To determine the administration dose, we carried out the dose-dependent experiment. In detail, 1 × 10^6^ B16-luc cells were implanted on the right flank of depilated C57BL/6 mice to establish tumor model of melanoma on day 0. On day 7, mice were divided into five groups (UnTx, 0.09 mg/kg, 0.18 mg/kg, 0.375 mg/kg, 0.75 mg/kg; the number of female C57BL/6 was 25) and were treated with different dose of small NPs intratumorally every 2 days. The tumor volumes were observed every 2 days. Tumor volume was calculated using the formulation: length × width × width/2.

The treatment efficiency of three different size of YCW NPs was evaluated on model of B16-luc tumor bearing C57BL/6 mice. 1 × 10^6^ B16-luc cells were implanted on the right flank of depilated C57BL/6 mice to establish tumor model of melanoma on day 0. On day 6, mice were divided into four groups (UnTx, large NPs, middle NPs, small NPs; the number of female C57BL/6 was 23) and were treated with 7.5 µg (50 µL per mouse, 0.375 mg/kg) of YCW NPs intratumorally every 2 days. To monitor the therapeutic effect of YCW NPs, the tumor volume and weight of tumor were observed every 2 days. Tumor volume was calculated using the formulation: length × width × width/2.

In experiment of treatment of B16-luc by combining small size of YCW NPs with anti-PD-L1, mice were divided into four groups (UnTx, small NPs, anti-PD-L1, combination; the number of female C57BL/6 was 20). The dosage and frequency of administration of small size of YCW NPs were the same as above. Anti-PD-L1 was injected intravenously every 3 days (50 µg per mouse). The volume of tumor and body weight of mice were recorded every 2 days. Hematoxylin-eosin staining (H&E staining) of major organs was used for assessing the safety of therapeutic. Mice were sacrificed on day 20 to analyze microenvironment of primary and distant tumor. The relative frequency of various immune cell subsets including T cells, MDSCs, Tregs, TAMs, as well as PD-L1 expression on tumor cells were examined by flow cytometry analysis.

In another treatment experiment, CT26 tumor cells (2 × 10^6^ per mouse) were injected at both sides of back of BALB/c mice. On day 6, small size YCW NPs and anti-PD-L1 were administrated as described above. The tumor volume and weight were monitored to evaluate the therapeutic effect. In lung metastasis model, 1 × 10^6^ B16-luc tumor cells were injected intravenously instead. The number of black spots representative of metastatic lesions in lung was counted to evaluate the treatment effect. The number of female BALB/c mice was 16.

To compare the treatment effect of our system (YCW NPs) and existing system (YCW MPs - GPs), we carried out therapeutic experiments with B16. 1 × 10^6^ B16-luc cells were implanted on the right flank of depilated C57BL/6 mice to establish tumor model of melanoma on day 0. On day 8, mice were divided into three groups (UnTx, YCW MPs – GPs, YCW small NPs; the number of female C57BL/6 was 12) and were treated with 7.5 µg (50 µL per mouse, 0.375 mg/kg) of GPs and NPs intratumorally every 2 days. To monitor the therapeutic effect, the tumor volume and weight of mice were observed every 2 days. Tumor volume was calculated using the formulation: length × width × width/2.

### Depletion of T cells

C57BL/6 mice were inoculated with B16-luc and divided into four groups (UnTx, anti-CD4 group, anti-CD8a group, no depletion group; the number of female C57BL/6 was 16). On day 4 and day 7, mice were injected intravenously with anti-CD4 (20 µg per mouse) and anti-CD8a (20 µg per mouse) respectively. Next, on day 8, peripheral blood mononuclear cells were collected to analyze the efficiency of T cell (including CD4 and CD8a) depletion. On the same day, the mice were treated with small size of YCW NPs every 2 days except for UnTx. The tumor size and weight were observed every 2 days.

### In vivo bioluminescence imaging

Tumor growth of B16-luc was monitored using in vivo IVIS Spectrum Imaging System (PerkinElmer Ltd). Mice were injected with D-luciferin (1.5 mg/mL) intraperitoneally, bioluminescence imaging was obtained after 10 min and exposure time was 30 s. Bioluminescence intensity was quantified as average radiance (photons s^−1^ cm^−2^ sr^−1^) with IVIS Living Image 4.2.

### Statistical analysis

All results were expressed as mean ± SD. Unless otherwise stated, all experiments used biological replication. Student’s *t* test (two-tailed) was utilized for two groups compared. One-way ANOVA using the Tukey post-test for more than two groups compared. All statistical analyses were performed using GraphPad Prism (8.0). For animal survival analysis, the statistical differences were determined by log-rank test. The results were significant when ****P < 0.0001; ***P < 0.005; **P < 0.01; *P < 0.05. All experiments were run at least in triplicate.

### Reporting summary

Further information on research design is available in the Nature Research Reporting Summary linked to this article.

## Supplementary information

Supplementary Information

Reporting Summary

## Data Availability

Source data are provided with this paper. The authors declare that all other data supporting the findings of this study are available within the paper, Supplementary Information or Source Data file. Source data are provided with this paper.

## Source data

Source Data
