# Supplementary material for: Yeast-derived nanoparticles remodel the immunosuppressive microenvironment in tumor and tumor-draining lymph nodes to suppress tumor growth
Source: Nat Commun. 2022 Jan 10;13:110. doi: 10.1038/s41467-021-27750-2 (PMC8748771; doi:10.1038/s41467-021-27750-2)
Supplement: Supplementary file 1 — Supplementary Information [file 41467_2021_27750_MOESM1_ESM.pdf]

Yeast-Derived Nanoparticles Remodel the Immunosuppressive Microenvironment in Tumor and Tumor-draining Lymph Nodes to Suppress Tumor Growth

Jialu Xu[1], Qingle Ma[1], Yue Zhang[1], Ziying Fei[1], Yifei Sun[2], Qin Fan[1], Bo Liu[1], Jinyu Bai[3], Yue Yu[1], Jianhong Chu[4]*, Jingrun Chen[2]*, Chao Wang[1]*

[1] Institute of Functional Nano & Soft Materials (FUNSOM), Jiangsu Key Laboratory for Carbon-based Functional Materials and Devices, Soochow University, Suzhou, Jiangsu 215123, China

[2] School of Mathematical Sciences, Soochow University, Suzhou, Jiangsu 215006, China

[3] The Second Affiliated Hospital of Soochow University, Suzhou, Jiangsu 215004, China

[4] Institute of Blood and Marrow Transplantation, Jiangsu Institute of Hematology, Collaborative Innovation Center of Hematology, National Clinical Research Center for Hematological Diseases, Soochow University, Suzhou, Jiangsu 215123, China

* Corresponding author: Chao Wang: cwang@suda.edu.cn, Jingrun Chen: jingrunchen@suda.edu.cn, Jianhong Chu: jhchu@suda.edu.cn

Supplementary Table 1 Materials used in study.

Supplementary Fig. 1 The composition of YCW NPs.

Supplementary Table 2 Specific information on the proteins contained in YCW NPs.

Supplementary Table 3 Specific information of mass spectrometry data of monosaccharide.

Supplementary Fig. 2 Characterization of YCW NPs with different sizes.

Supplementary Fig. 3 In vitro cytotoxicity assessment evaluated by MTT assay.

Supplementary Fig. 4 Activation of BMDCs and RAW264.7 after incubation with YCW NPs with different sizes.

Supplementary Fig. 5 Activation of BMDCs induced by YCW particles.

Supplementary Fig. 6 Flow cytometry gating strategy for analysis of BMDCs maturation.

Supplementary Fig. 7 Complete initial data of western blotting analysis of Dectin-1 / Syk pathway and TLR2 / MyD88 pathway.

Supplementary Fig. 8 The influence of YCW NPs on T cells in vitro.

Supplementary Fig. 9 Dose-response experiment for antitumor response.

Supplementary Fig. 10 YCW NPs inhibited tumor growth by remodeling immunosuppressive tumor microenvironment.

Supplementary Fig. 11 Flow cytometry gating strategy for analysis of different cells in tumor.

Supplementary Fig. 12 Distribution of YCW micro-particles (MPs).

Supplementary Fig. 13 Distribution of YCW NPs in vivo.

Supplementary Fig. 14 Flow cytometry gating strategy for analysis of T cells in blood.

Supplementary Fig. 15 H&E staining and flow cytometry analysis of TME after therapeutic.

Supplementary Fig. 16 Combination therapeutic with small size YCW NPs and anti-PD-L1 for B16.

Supplementary Fig. 17 Treatment efficiency of YCW small NPs and YCW MPs (GPs).

Supplementary Table 1. Materials used in study.

Antibodies	Manufacturer	Catalog Number	Dilutions
FITC anti-mouse CD3	Biolegend	100204	1:200
APC anti-mouse CD4	Biolegend	100412	1:200
PE anti-mouse CD8a	Biolegend	100708	1:200
FITC anti-mouse CD11c	Biolegend	117306	1:200
APC anti-mouse Ly-6G/Ly-6C (Gr-1)	Biolegend	108412	1:200
FITC anti-mouse F4/80	Biolegend	123108	1:200
PE anti-mouse CD206 (MMR)	Biolegend	141706	1:200
FITC anti-mouse CD45	Biolegend	103108	1:200
PerCP anti-mouse/human CD11b	Biolegend	101230	1:200
PE anti-mouse FOXP3	Biolegend	126403	1:200
APC anti-mouse CD80	Biolegend	104714	1:200
PE anti-mouse CD86	Biolegend	105008	1:200
PE anti-mouse CD274 (B7-H1, PD-L1)	Biolegend	124308	1:200
APC anti-mouse CD279 (PD-1)	Biolegend	135209	1:200
PE anti-mouse I-A/I-E	Biolegend	107608	1:200
APC anti-mouse CD40	Biolegend	124612	1:200
APC anti-mouse CD69	Biolegend	104514	1:200
PE anti-mouse CD19	Biolegend	15508	1:200
Anti-CD4	BioXcell	BE0004-1;<br>Clone: 53-6.7	Diluted to 100 g/mL
Anti-CD8a	BioXcell	BE0003-1;<br>Clone: GK1.5	Diluted to 100 g/mL
Anti-PD-L1	BioXcell	BE0101;<br>Clone: 10F. 9G2;<br>Lot: 751219D1	Diluted to 100 g/mL
Anti-p-P65	Abclonal	AP0475	1:1000
Anti-GAPDH	Servicebio	GB11002	1:1000
Anti-TLR-2	Abcam	ab209216	1:1000
Anti-p-Syk	Cell Signaling Technology	#2710	1:1000
Anti-MyD88	Abcam	ab219413	1:1000
Anti-Dectin-1	Absin	abs124190	1:1000
Goat anti-Rabbit IgG (H+L)-HRP	Ray Antibody	RM3002	1:5000

**Yeast**			
Yeast from *Saccharomyces Cerevisiae*	Sigma-aldrich	ysc2-500g	
**Inhibitor**			
Laminarin	Sigma-aldrich	9008-22-4	
C29	Cayman	27029	

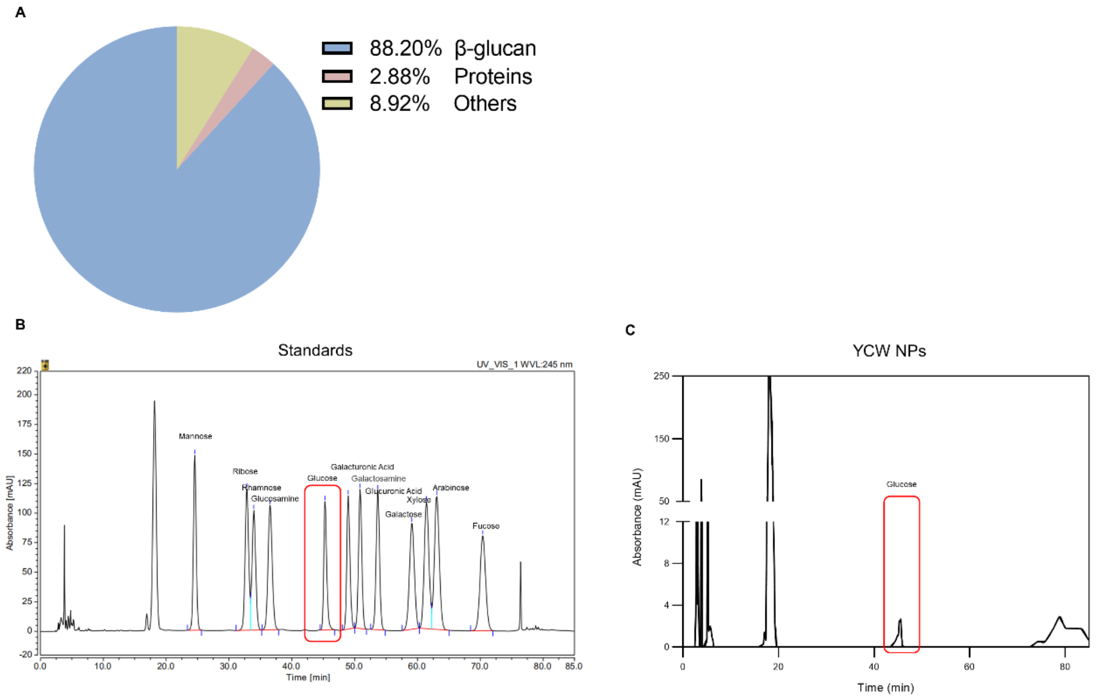

Supplementary Fig. 1. The composition of YCW NPs. (**A**) The composition of YCW nanoparticles. (**B**) Monosaccharide standard high performance liquid chromatography (HPLC) curve. (**C**) Monosaccharide curve of YCW NPs.

Protein Group	Protein ID	Accession	-10lgP	PTM	Avg. Mass	Description
23	15	P60010 ACT_YEAST	156.24		41690	Actin OS=Saccharomyces cerevisiae (strain ATCC 204508 / S288c) OX=559292 GN=ACT1 PE=1 SV=1
20	24	P07251 ATPA_YEAST	139.36	Oxidation (HW)	58608	ATP synthase subunit alpha mitochondrial OS=Saccharomyces cerevisiae (strain ATCC 204508 / S288c) OX=559292 GN=ATP1 PE=1 SV=5
12	42	P02992 EFTU_YEAST	116.47	Acetylation (N-term);Oxidation (HW)	47972	Elongation factor Tu mitochondrial OS=Saccharomyces cerevisiae (strain ATCC 204508 / S288c) OX=559292 GN=TUF1 PE=1 SV=1
30	50	P05030 PMA1_YEAST	110.87	Carbamidomethylation;Oxidation (HW)	99619	Plasma membrane ATPase 1 OS=Saccharomyces cerevisiae (strain ATCC 204508 / S288c) OX=559292 GN=PMA1 PE=1 SV=2
35	105	P00925 ENO2_YEAST	101.23		46914	Enolase 2 OS=Saccharomyces cerevisiae (strain ATCC 204508 / S288c) OX=559292 GN=ENO2 PE=1 SV=2
35	113	P00924 ENO1_YEAST	101.23		46816	Enolase 1 OS=Saccharomyces cerevisiae (strain ATCC 204508 / S288c) OX=559292 GN=ENO1 PE=1 SV=3
31	66	P00331 ADH2_YEAST	96.07	Carbamidomethylation;Acetylation (N-term)	36732	Alcohol dehydrogenase 2 OS=Saccharomyces cerevisiae (strain ATCC 204508 / S288c) OX=559292 GN=ADH2 PE=1 SV=3
31	36	P00330 ADH1_YEAST	96.07	Carbamidomethylation;Acetylation (N-term);Oxidation (HW)	36849	Alcohol dehydrogenase 1 OS=Saccharomyces cerevisiae (strain ATCC 204508 / S288c) OX=559292 GN=ADH1 PE=1 SV=5
37	252	P00830 ATPB_YEAST	95.58		54794	ATP synthase subunit beta mitochondrial OS=Saccharomyces cerevisiae (strain ATCC 204508 / S288c) OX=559292 GN=ATP2 PE=1 SV=2
38	160	P39954 SAHH_YEAST	89.76	Carbamidomethylation	49126	Adenosylhomocysteinase OS=Saccharomyces cerevisiae (strain ATCC 204508 / S288c) OX=559292 GN=SAH1 PE=1 SV=1
32	168	P02994 EF1A_YEAST	88.56		50033	Elongation factor 1-alpha OS=Saccharomyces cerevisiae (strain ATCC 204508 / S288c) OX=559292 GN=TEF2 PE=1 SV=1
40	99	P02309 H4_YEAST	87.2		11368	Histone H4 OS=Saccharomyces cerevisiae (strain ATCC 204508 / S288c) OX=559292 GN=HHF2 PE=1 SV=2
34	138	P00549 KPYK1_YEAST	84.34	Carbamidomethylation	54545	Pyruvate kinase 1 OS=Saccharomyces cerevisiae (strain ATCC 204508 / S288c) OX=559292 GN=CDC19 PE=1 SV=2
36	98	P00560 PGK_YEAST	83.01		44738	Phosphoglycerate kinase OS=Saccharomyces cerevisiae (strain ATCC 204508 / S288c) OX=559292 GN=PGK1 PE=1 SV=2
41	1031	Q12692 H2AZ_YEAST	71.6		14283	Histone H2A.Z OS=Saccharomyces cerevisiae (strain ATCC 204508 / S288c) OX=559292 GN=HTZ1 PE=1 SV=3
56	335	P05750 RS3_YEAST	58.02		26503	40S ribosomal protein S3 OS=Saccharomyces cerevisiae (strain ATCC 204508 / S288c) OX=559292 GN=RPS3 PE=1 SV=5
48	204	P39708 DHE5_YEAST	52.46	Oxidation (HW)	49627	NADP-specific glutamate dehydrogenase 2 OS=Saccharomyces cerevisiae (strain ATCC 204508 / S288c) OX=559292 GN=GDH3 PE=3 SV=1
48	293	P07262 DHE4_YEAST	52.46		49570	NADP-specific glutamate dehydrogenase 1 OS=Saccharomyces cerevisiae (strain ATCC 204508 / S288c) OX=559292 GN=GDH1 PE=1 SV=2
53	2043	P25515 VATL1_YEAST	51.44	Carbamidomethylation	16351	V-type proton ATPase subunit c OS=Saccharomyces cerevisiae (strain ATCC 204508 / S288c) OX=559292 GN=VMA3 PE=1 SV=1
60	6467	Q08692 OSW1_YEAST	50.67		32758	Outer spore wall protein 1 OS=Saccharomyces cerevisiae (strain ATCC 204508 / S288c) OX=559292 GN=OSW1 PE=4 SV=1
44	278	P22202 HSP74_YEAST	48.58	Acetylation (N-term)	69651	Heat shock protein SSA4 OS=Saccharomyces cerevisiae (strain ATCC 204508 / S288c) OX=559292 GN=SSA4 PE=1 SV=3
44	127	P09435 HSP73_YEAST	48.58	Acetylation (N-term)	70547	Heat shock protein SSA3 OS=Saccharomyces cerevisiae (strain ATCC 204508 / S288c) OX=559292 GN=SSA3 PE=1 SV=3
47	74	P00360 G3P1_YEAST	44.68	Carbamidomethylation	35750	Glyceraldehyde-3-phosphate dehydrogenase 1 OS=Saccharomyces cerevisiae (strain ATCC 204508 / S288c) OX=559292 GN=TDH1 PE=1 SV=3
55	226	P80210 PURA_YEAST	43.86		48279	Adenylosuccinate synthetase OS=Saccharomyces cerevisiae (strain ATCC 204508 / S288c) OX=559292 GN=ADE12 PE=1 SV=3
42	180	Q06108 RGC1_YEAST	42.56		120395	Regulator of the glycerol channel 1 OS=Saccharomyces cerevisiae (strain ATCC 204508 / S288c) OX=559292 GN=RGC1 PE=1 SV=1
49	283	P15179 SYDM_YEAST	41.88		75461	Aspartate--tRNA ligase mitochondrial OS=Saccharomyces cerevisiae (strain ATCC 204508 / S288c) OX=559292 GN=MSD1 PE=1 SV=1
45	2778	P53312 SUCB_YEAST	41.27		46901	Succinate--CoA ligase [ADP-forming] subunit beta mitochondrial OS=Saccharomyces cerevisiae (strain ATCC 204508 / S288c) OX=559292 GN=LSC2 PE=1 SV=1
46	86	P19882 HSP60_YEAST	41.23		60752	Heat shock protein 60 mitochondrial OS=Saccharomyces cerevisiae (strain ATCC 204508 / S288c) OX=559292 GN=HSP60 PE=1 SV=1
51	1996	P38156 MAL31_YEAST	40.55		68263	Maltose permease MAL31 OS=Saccharomyces cerevisiae (strain ATCC 204508 / S288c) OX=559292 GN=MAL31 PE=1 SV=1
54	985	P02294 H2B2_YEAST	39.98		14237	Histone H2B.2 OS=Saccharomyces cerevisiae (strain ATCC 204508 / S288c) OX=559292 GN=HTB2 PE=1 SV=2

Supplementary Table 2. Specific information on the proteins contained in YCW NPs.

Number		Retention Time (min)	Peak Area (mAU*min)	Peak Hight (mAU)	Relative peak area (%)	Relative peak height (%)	Sample Amount (ug/g)
1	Mannose	24.547	76.931	147.967	8.17	11.20	30.5912
2	Ribose	32.843	80.046	119.705	8.50	9.06	30.7059
3	Rhamnose	33.957	68.725	101.261	7.30	7.67	37.8932
4	Glucosamine	36.543	81.359	105.265	8.64	7.97	36.1403
5	Glucose	45.287	58.693	108.620	6.23	8.22	29.5305
6	Galacturonic Acid	48.975	64.962	112.869	6.90	8.54	32.1512
7	Galactosamine	50.873	71.417	117.822	7.58	8.92	29.5746
8	Glucuronic Acid	53.685	75.380	117.666	8.00	8.91	40.5044
9	Galactose	59.113	82.683	89.621	8.78	6.78	32.4719
10	Xylose	61.437	94.660	107.369	10.05	8.13	33.4831
11	Arabinose	63.068	103.582	112.464	11.00	8.51	35.7434
12	Fucose	70.398	83.388	80.295	8.85	6.08	31.5859
Total			941.827	1320.923	100.00	100.00	

Number		Retention Time (min)	Peak Area (mAU*min)	Peak Hight (mAU)	Relative peak area (%)	Relative peak height (%)	Sample Amount (ug/g)
n.a.	Mannose	n.a.	n.a.	n.a.	n.a.	n.a.	n.a.
n.a.	Ribose	n.a.	n.a.	n.a.	n.a.	n.a.	n.a.
n.a.	Rhamnose	n.a.	n.a.	n.a.	n.a.	n.a.	n.a.
n.a.	Glucosamine	n.a.	n.a.	n.a.	n.a.	n.a.	n.a.
1	Glucose	45.340	2.006	2.157	100.0	100.00	221.1620
n.a.	Galacturonic Acid	n.a.	n.a.	n.a.	n.a.	n.a.	n.a.
n.a.	Galactosamine	n.a.	n.a.	n.a.	n.a.	n.a.	n.a.
n.a.	Glucuronic Acid	n.a.	n.a.	n.a.	n.a.	n.a.	n.a.
n.a.	Galactose	n.a.	n.a.	n.a.	n.a.	n.a.	n.a.
n.a.	Xylose	n.a.	n.a.	n.a.	n.a.	n.a.	n.a.
n.a.	Arabinose	n.a.	n.a.	n.a.	n.a.	n.a.	n.a.
n.a.	Fucose	n.a.	n.a.	n.a.	n.a.	n.a.	n.a.
Total			2.006	2.157	100.00	100.00	

Supplementary Table 3. Specific information of mass spectrometry data of monosaccharide. Standards (up) and YCW NPs (down).

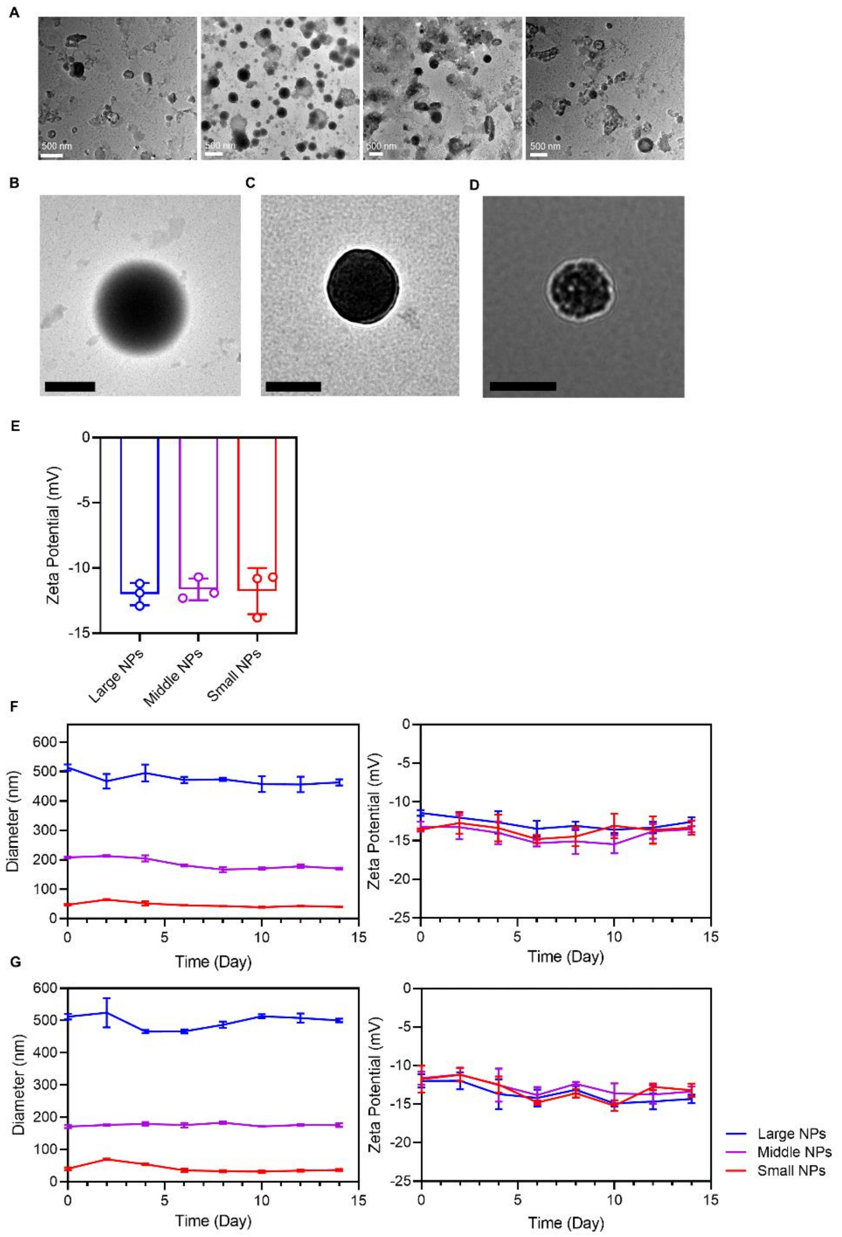

Supplementary Fig. 2 (related to Figure 1). Characterization of YCW NPs with different sizes. (**A**) TEM of mixture nanoparticles (Scale bar = 500 nm). We found that most of the broken particles were spherical or quasi-spherical, whereas NPs with irregular shape were also observed. (**B**) TEM of large size of YCW NPs (Scale bar = 500 nm). (**C**) TEM of middle size of YCW NPs (Scale bar = 100 nm). (**D**) TEM of small size of YCW NPs (Scale bar = 50 nm). (**E**) Zeta potential of YCW NPs with different sizes (n = 3). (**F-G**) Diameter and zeta potential of three different size of YCW NPs within two weeks to monitor the stability of YCW NPs (n = 3). (**F**) RT. (**G**) 4℃. Data are means ± SD.

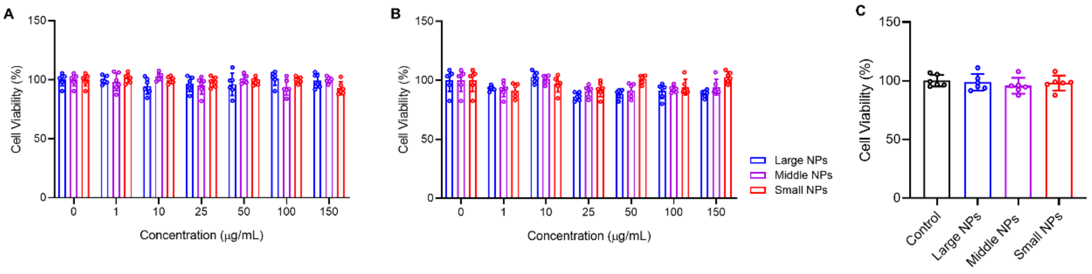

Supplementary Fig. 3. In vitro cytotoxicity assessment evaluated by MTT assay. (**A**) Dendritic cells. (**B**) Macrophages. (**C**) B16 (the concentration of YCW NPs was 150 μg/mL) (n=6). Data are means ± SD.

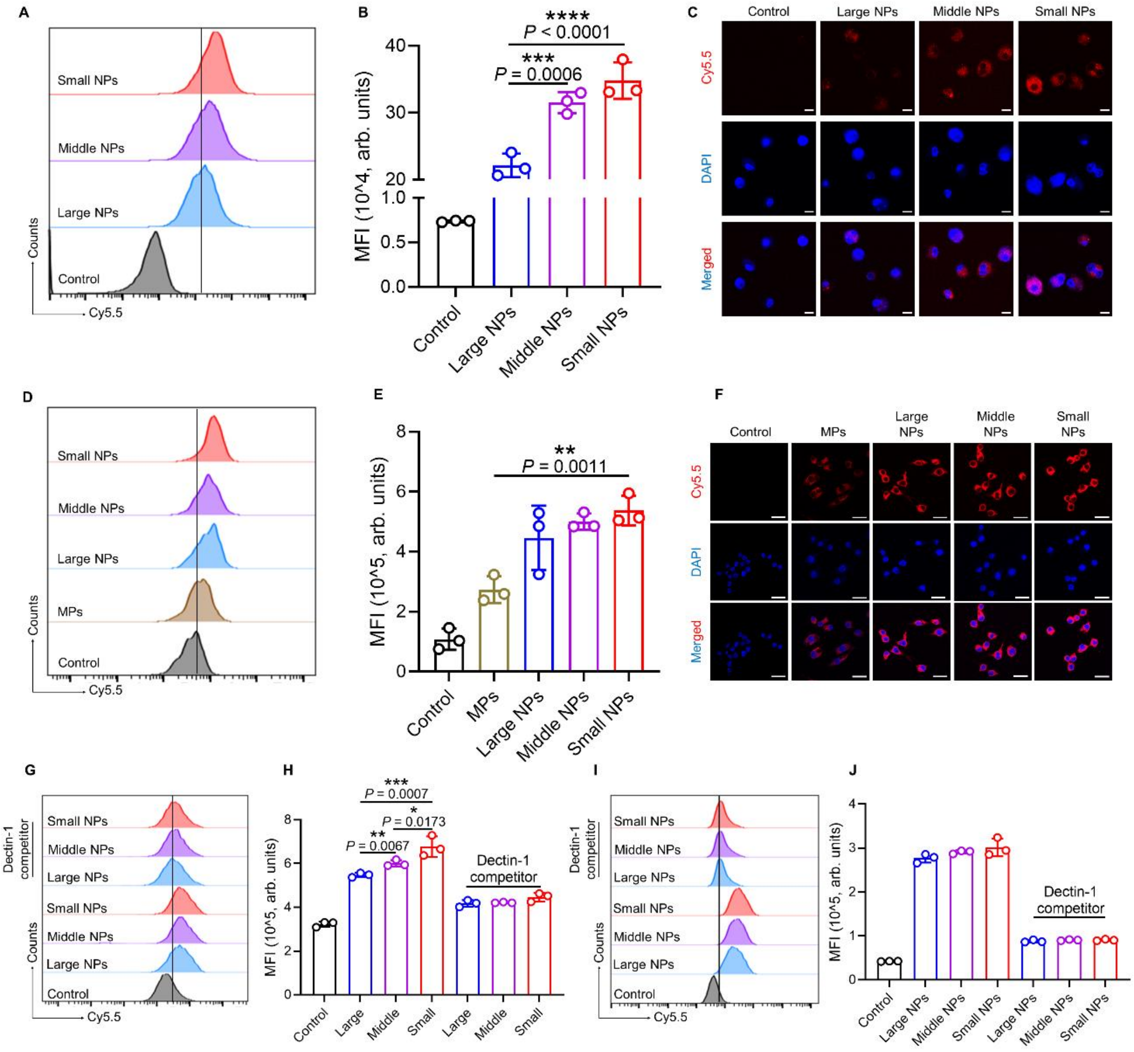

Supplementary Fig. 4. Activation of BMDCs and RAW264.7 after incubation with YCW NPs with different sizes. (**A**) Representative flow cytometric analysis of Cy5.5 expression and (**B**) corresponding quantitative analysis of Cy5.5 expression on BMDCs after incubation Cy5.5-labelled YCW NPs with different sizes for 24 h (n = 3). (**C**) Confocal imaging of Cy5.5-labelled YCW particles with different sizes after incubation with BMDCs for 24 h (blue: DAPI; red: Cy5.5; Scale bar = 10 μm, n = 3). (**D**) Representative flow cytometric analysis of Cy5.5 expression on RAW264.7 after incubation Cy5.5-labelled YCW particles (including MPs, Large NPs, Middle NPs, Small NPs) for 24 h. (**E**) The corresponding quantification of Cy5.5 (MFI) after RAW264.7 incubation with Cy5.5-labelled particles for 24 h (n = 3). (**F**) Confocal imaging of Cy5.5-labelled YCW particles with different sizes after incubation with RAW264.7 for 24 h (blue: DAPI; red: Cy5.5; Scale bar = 20 μm, n = 3). (**G**) Representative flow cytometric analysis and (**H**) corresponding quantification of Cy5.5 (MFI) after incubation of DC2.4 with Cy5.5-labelled YCW particles for 24 h (n = 3). (**I**)

Representative flow cytometric analysis and (**J**) corresponding quantification of Cy5.5 (MFI) after incubation of RAW264.7 with Cy5.5-labelled YCW particles for 24 h (n = 3). Statistical significance was obtained by one-way ANOVA using the Tukey post-test. **** $P < 0.0001$; *** $P < 0.005$; ** $P < 0.01$; * $P < 0.05$. Data are means ± SD. MFI: mean fluorescence intensity; arb. units: arbitrary units.

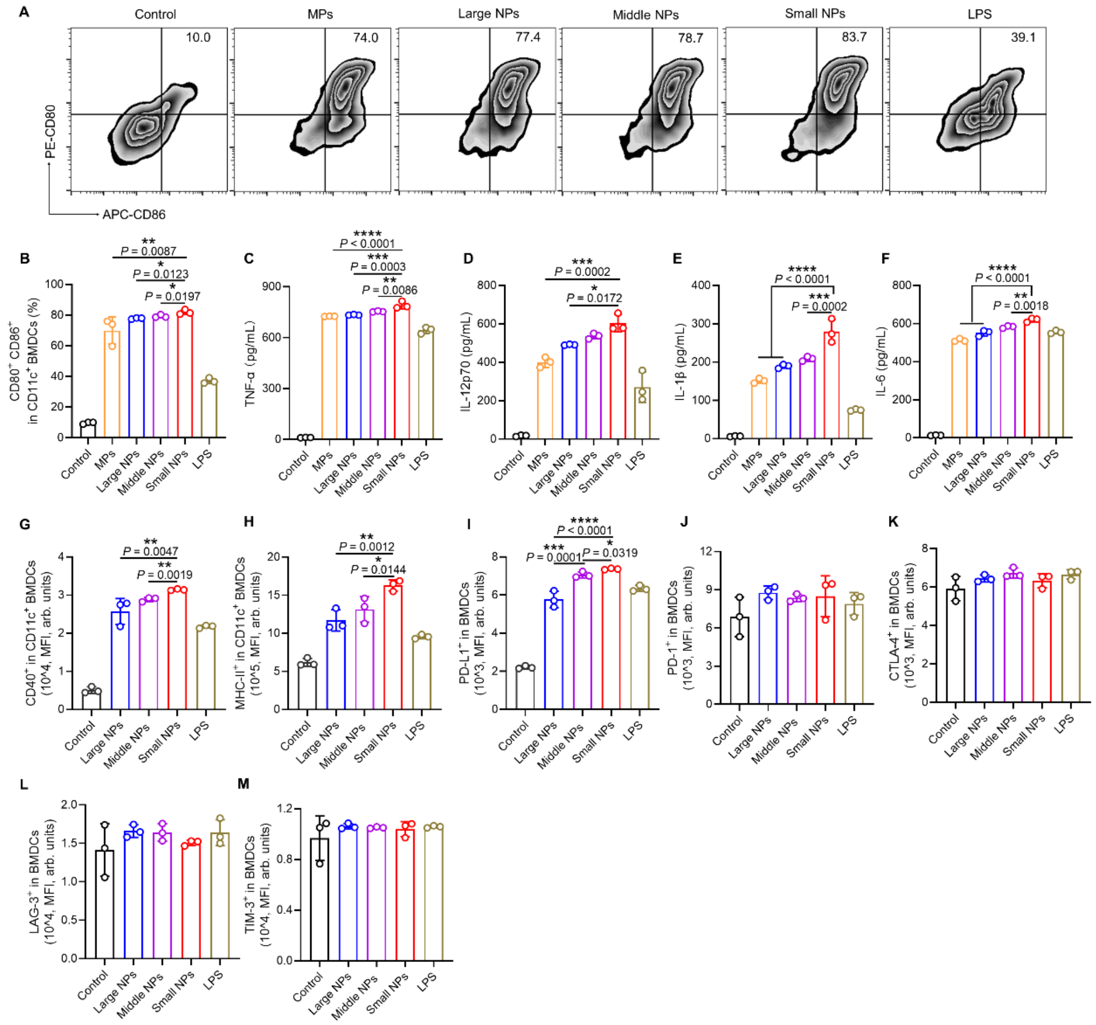

Supplementary Fig. 5 (related to Figure 2). Activation of BMDCs induced by YCW particles. (**A-B**) Activation of BMDCs after BMDCs incubation with YCW particles, including MPs, large NPs, middle NPs, small NPs and LPS (positive control) for 24 h. (**A**) Representative dot plots of co-stimulatory molecules CD80 and CD86 expression on BMDCs and (**B**) corresponding quantification of BMDCs maturation (n = 3). (**C-F**) Concentration of pro-inflammatory cytokines secreted by BMDCs after incubation with YCW particles as indicated. (**C**) TNF-α; (**D**) IL-12p70; (**E**) IL-1β; (**F**) IL-6 (n = 3). (**G**) The corresponding quantification of CD40 expression on BMDCs after YCW NPs incubation with BMDCs for 24 h (n = 3). (**H**) The corresponding quantification of MHC Ⅱ expression on BMDCs after YCW NPs incubation with BMDCs for 24 h (n = 3). (**I**) The corresponding quantification of PD-L1 expression on BMDCs after YCW NPs incubation with BMDCs for 24 h (n = 3). (**J**) The corresponding quantification of PD-1 expression on BMDCs after YCW NPs incubation with BMDCs for 24 h (n = 3). (**K**) The corresponding quantification of CTLA-4 expression on

BMDCs after YCW NPs incubation with BMDCs for 24 h (n = 3). (**L**) The corresponding quantification of LAG-3 expression on BMDCs after YCW NPs incubation with BMDCs for 24 h (n = 3). (**M**) The corresponding quantification of TIM-3 expression on BMDCs after YCW NPs incubation with BMDCs for 24 h (n = 3). Statistical significance was obtained by one-way ANOVA using the Tukey post-test. **** $P < 0.0001$; *** $P < 0.005$; ** $P < 0.01$; * $P < 0.05$. Data are means ± SD. MFI: mean fluorescence intensity; arb. units: arbitrary units.

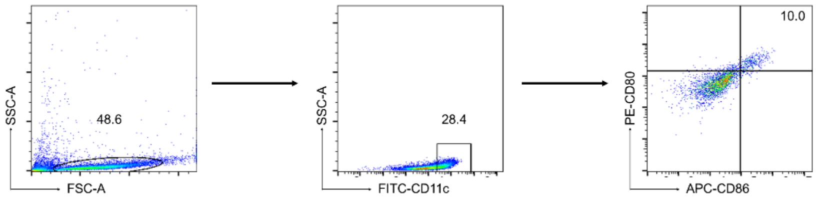

Supplementary Fig. 6 (related to Figure 2). Flow cytometry gating strategy for analysis of BMDCs maturation.

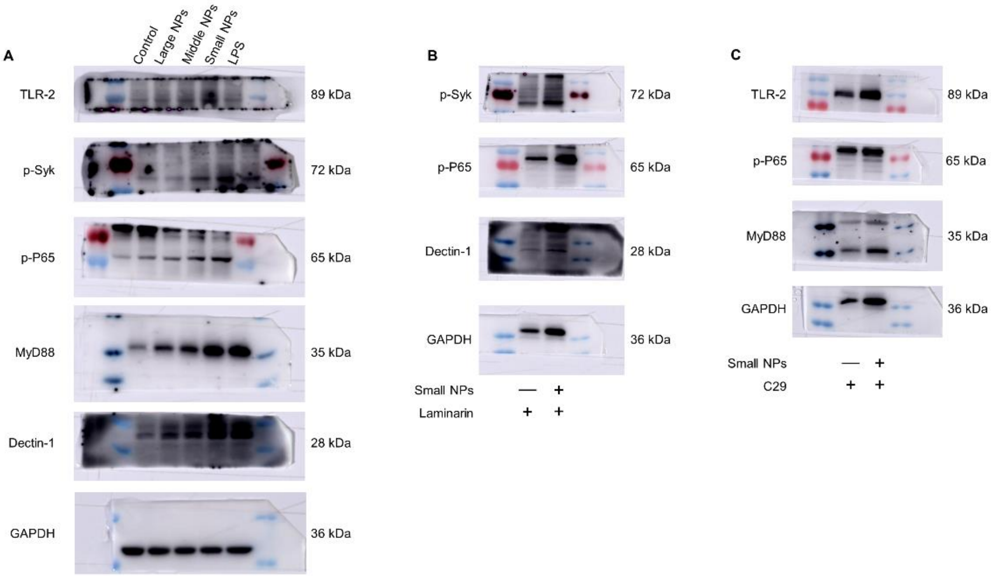

Supplementary Fig. 7 (related to Figure 2). Complete initial data of western blotting analysis of Dectin-1 / Syk pathway and TLR2 / MyD88 pathway. (**A**)Western blotting analysis of Dectin-1 / Syk pathway and TLR2 / MyD88 pathway from proteins of BMDCs after incubation with three YCW NPs and LPS for 24 h, including TLR2, p-Syk, p-P65, MyD88, Dectin-1. (**B**) After utilizing Dectin-1 competitor laminarin for 2 h, western blotting analysis of Dectin-1 / Syk pathway from proteins of BMDCs after incubation with Small NPs for 24 h, including p-Syk, p-P65, Dectin-1. (**C**) After utilizing TLR2 inhibitor C29 for 2 h, western blotting analysis of TLR2 / MyD88 pathway from proteins of BMDCs after incubation with Small NPs for 24 h, including p-P65, TLR2, MyD88 (n = 3, each experiment was repeated three times independently with similar results, and we chose one results to show).

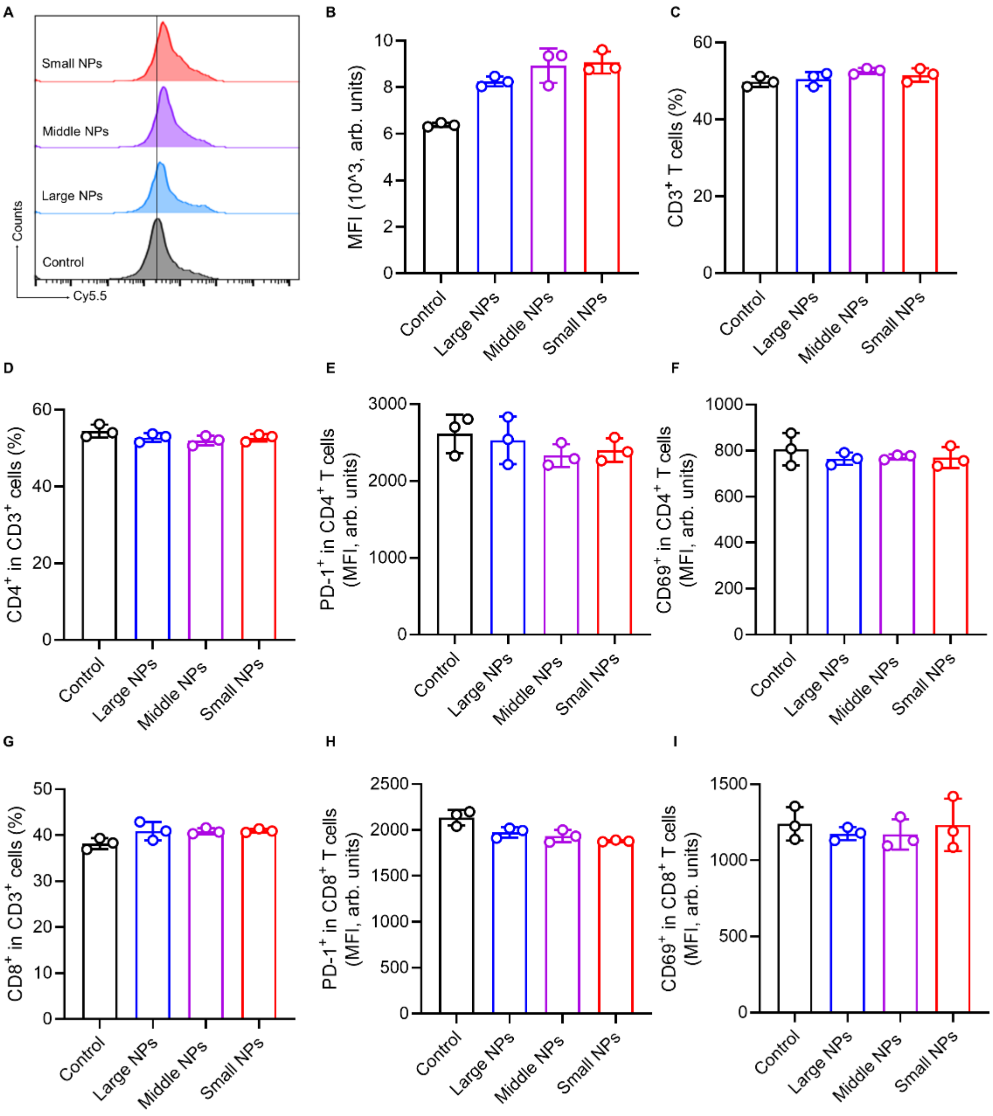

Supplementary Fig. 8. The influence of YCW NPs on T cells in vitro. (**A**) Representative flow cytometric analysis and (**B**) corresponding quantitative analysis of Cy5.5 intensity on T cells after incubation of T cells with Cy5.5-labelled YCW NPs (including large NPs, middle NPs, small NPs) for 24 h (n = 3). (**C-I**) Corresponding quantitative analysis of CD3 T cells (**C**); CD4$^+$ cells in CD3 cells (**D**); PD-1 expression on CD4$^+$ T cells (**E**); CD69 expression on CD4$^+$ T cells (**F**); CD8 expression on CD3 cells (**G**); PD-1 expression on CD8$^+$ T cells (**H**); CD69 expression on CD8$^+$ T cells (**I**) (n = 3). Statistical significance was obtained by one-way ANOVA using the Tukey post-test. Data are means ± SD. MFI: mean fluorescence intensity; arb. units: arbitrary units.

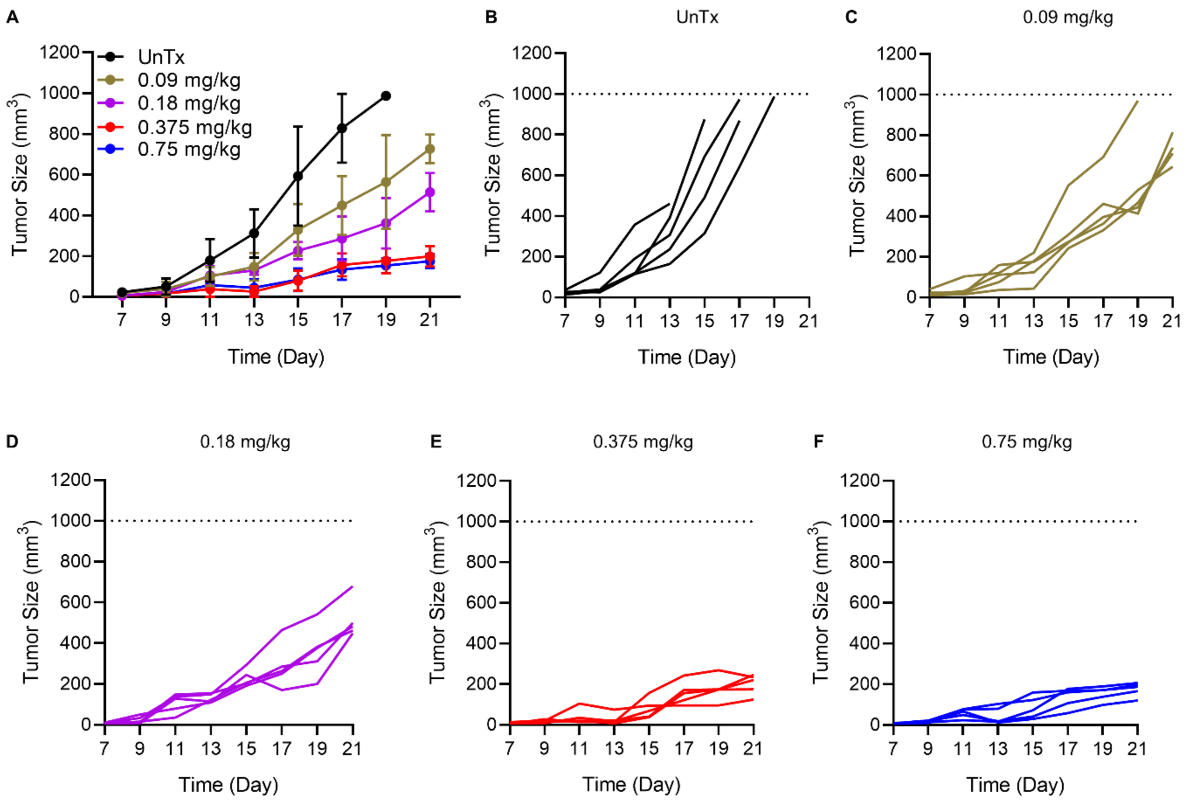

Supplementary Fig. 9. Dose-response experiment for antitumor response. (**A**) Average and (**B-F**) individual tumor growth curves in different dose group, including 0 mg/kg, 0.09 mg/kg, 0.18 mg/kg, 0.375 mg/kg and 0.75 mg/kg (n = 5). Data are means ± SD.

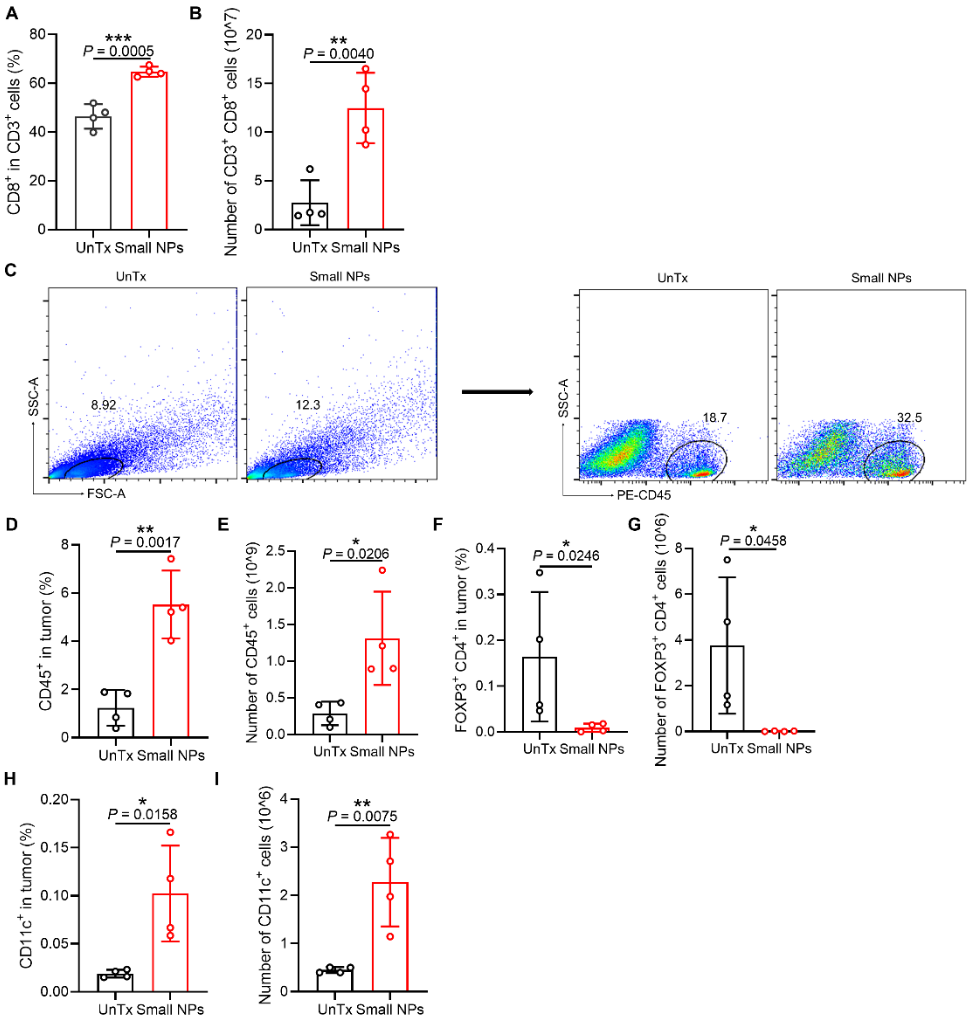

Supplementary Fig. 10 (related to Figure 3). YCW NPs inhibited tumor growth by remodeling immunosuppressive tumor microenvironment. (**A**) Proportion of $CD8^+$ T cells in $CD3^+$ T cells. (**B**) Absolute numbers of the $CD8^+$ of the tumor in UnTx group and small NPs group. (**C**) Representative flow cytometric analysis for $CD45^+$ in tumors and (**D**) corresponding quantitative analysis of untreated group and Small NPs group. (**E**) Absolute numbers of the $CD45^+$ of the tumor. (**F**) Corresponding quantitative analysis for $FOXP3^+$ $CD4^+$ in tumors of untreated group and small NPs group. (**G**) Absolute numbers of the $CD4^+$ $FOXP3^+$ of the tumor. (**H**) Corresponding quantitative analysis of untreated group and Small NPs group. (**I**) Absolute numbers of the $CD11c^+$ of the tumor (n = 4). Statistical significance was obtained by Student's t tests (two-tailed). *** $P < 0.005$; ** $P < 0.01$; * $P < 0.05$. Data are means ± SD.

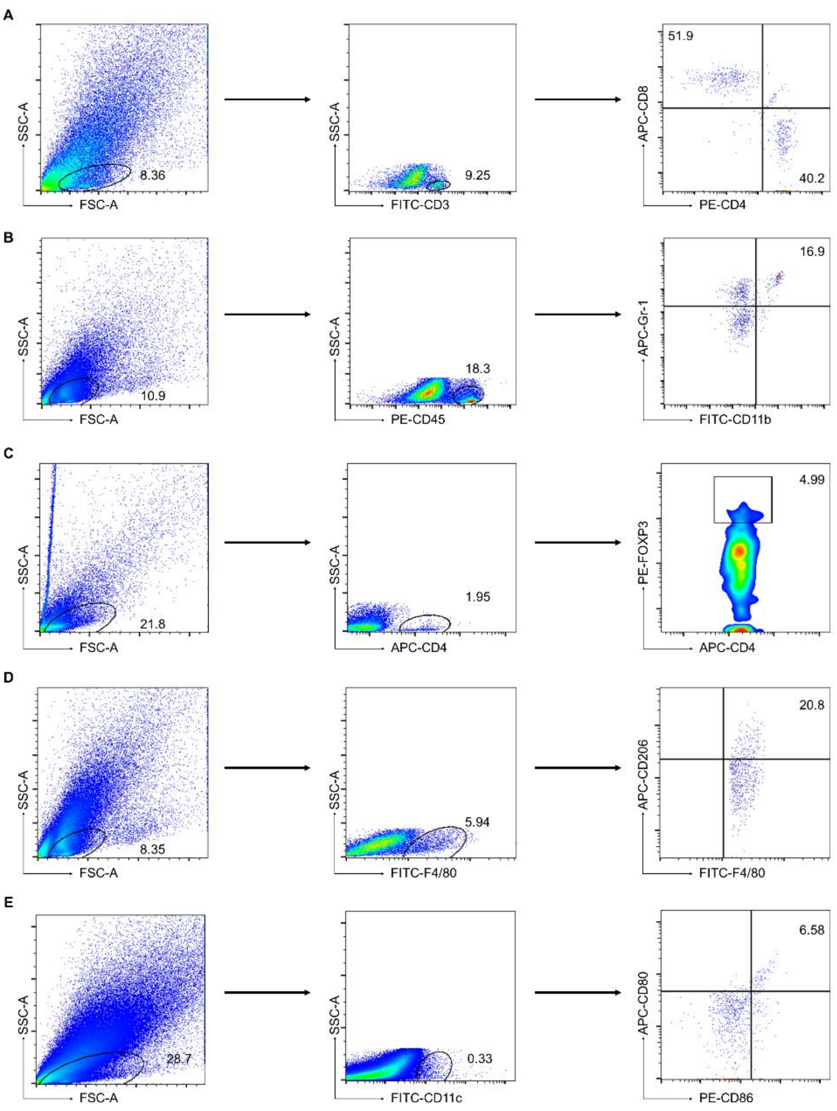

Supplementary Fig. 11 (related to Figure 3). Flow cytometry gating strategy for analysis of different cells in tumor. (**A**) T cells; (**B**) MDSCs; (**C**) Tregs; (**D**) TAMs; (**E**) DC maturation.

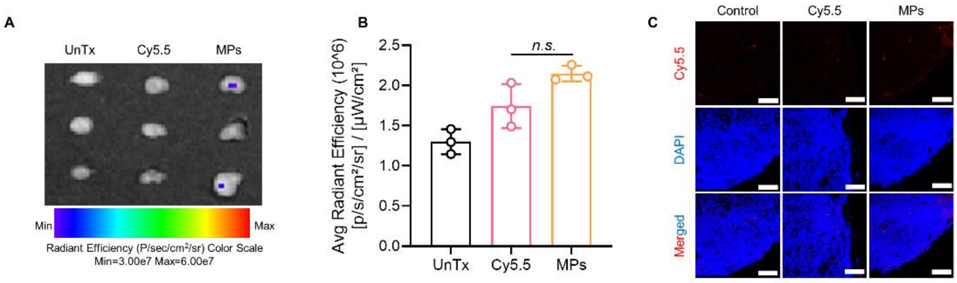

Supplementary Fig. 12. Distribution of YCW micro-particles (MPs). (**A**) Fluorescence imaging of TDLNs ex vivo after injection of Cy5.5 and Cy5.5-labelled MPs for 48 h. (**B**) Signal quantification of Cy5.5-labelled MPs in TDLNs (n = 3). (**C**) Confocal imaging of TDLNs to monitor the signal of Cy5.5 (blue: DAPI; red: Cy5.5; Scale bar = 80 µm, n = 3). Statistical significance was obtained by one-way ANOVA using the Tukey post-test. Data are means ± SD.

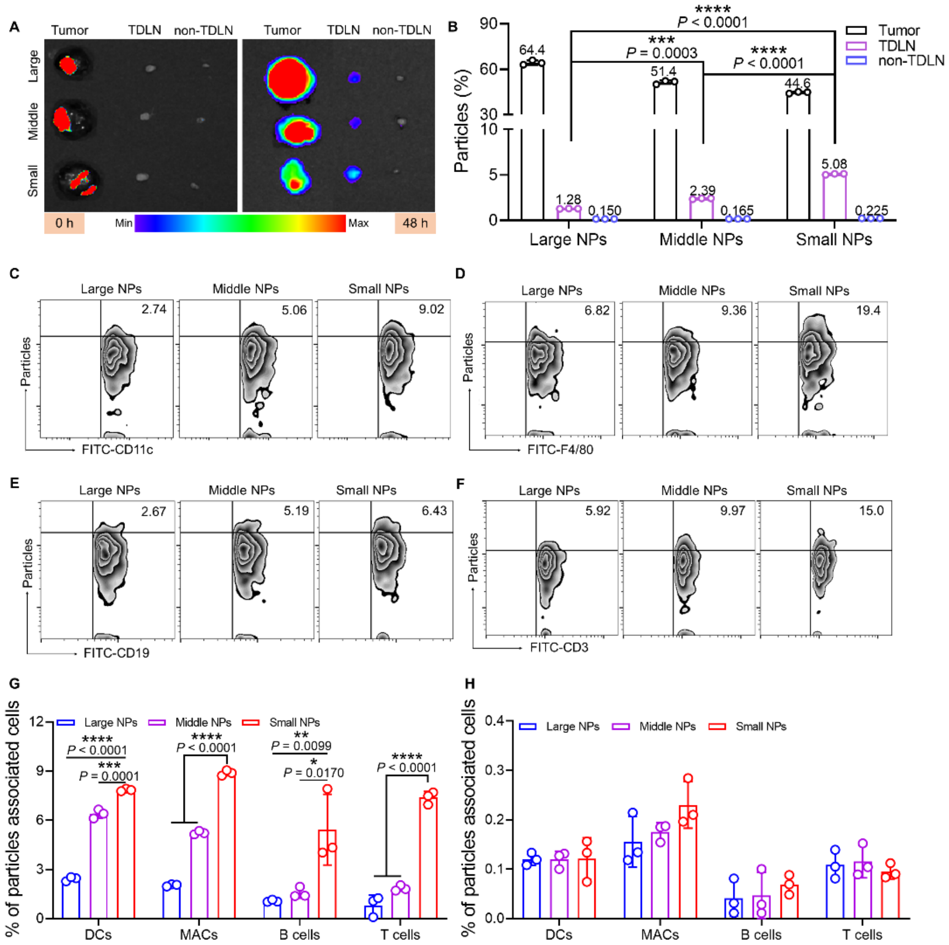

Supplementary Fig. 13 (related to Figure 4). Distribution of YCW NPs in vivo. (**A**) Fluorescence imaging of tumor, TDLN and non-TDLN ex vivo after injection of Cy5.5-labelled NPs for 48 h. (**B**) Proportions of NPs in tumor, TDLN and non-TDLN after injection of Cy5.5-labelled NPs for 48 h (n = 3). (**C-F**) Representative flow cytometric analysis of immune cells with TDLNs after injection of Cy5.5-labelled YCW NPs for 48 h. (**C**) DCs; (**D**) Macrophages; (**E**) B cells; (**F**) T cells. (**G**) Proportions of particles associated cells of tumor after intratumorally injection for 48 h (n = 3). (**H**) Proportions of particles associated cells of non-tumor draining lymph nodes after intratumorally injection for 48 h (n = 3). Statistical significance was obtained by one-way ANOVA using the Tukey post-test. **** *P* < 0.0001; *** *P* < 0.005; ** *P* < 0.01; * *P* < 0.05. Data are means ± SD.

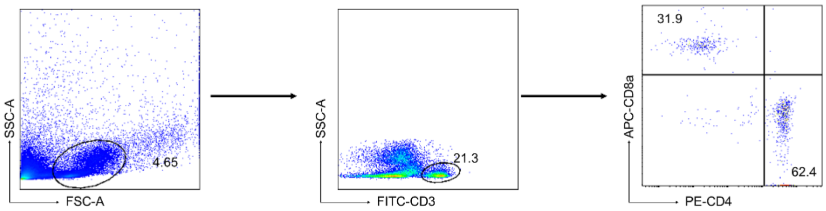

Supplementary Fig. 14 (related to Figure 5). Flow cytometry gating strategy for analysis of T cells in blood.

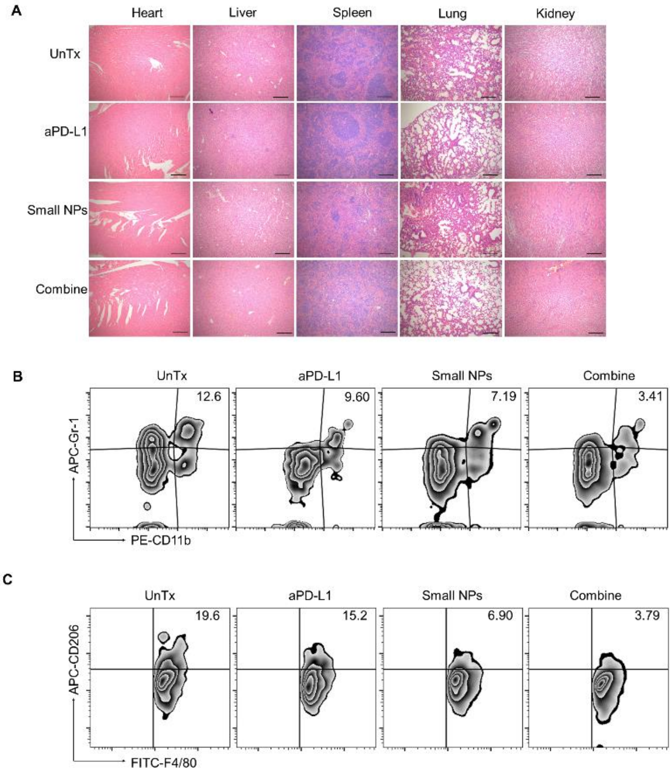

Supplementary Fig. 15 (related to Figure 6). H&E staining and flow cytometry analysis of TME after therapeutic. (**A**) H&E staining of major organs of each group after combination therapy (Scale bar = 200 µm, n = 3). (**B**) Representative flow cytometry analysis of MDSCs and (**C**) representative flow cytometry analysis of TAMs within the tumors.

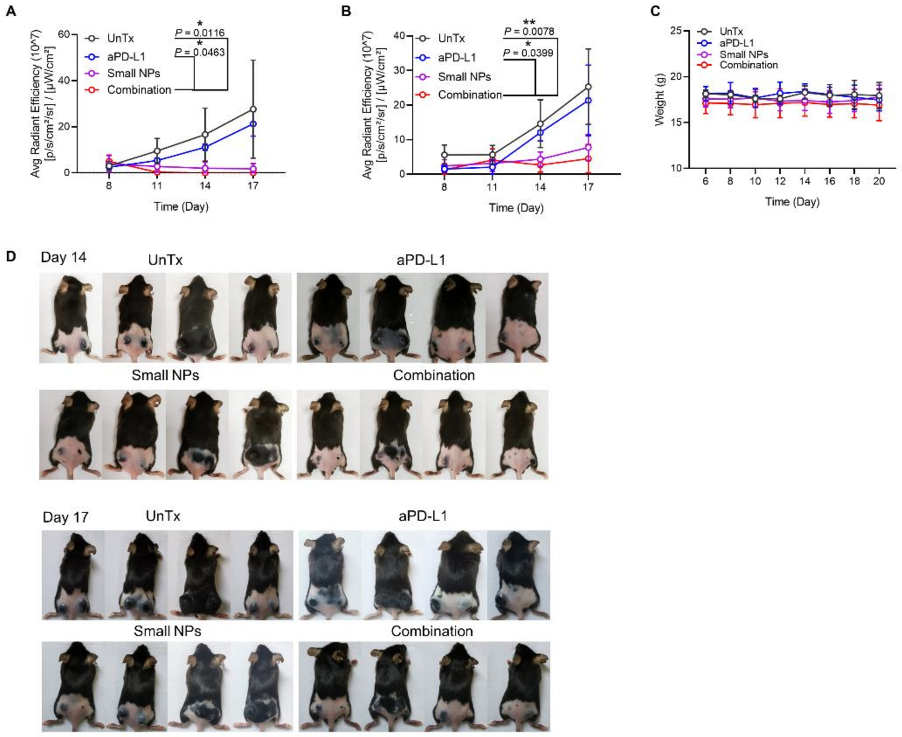

Supplementary Fig. 16 (related to Figure 7). Combination therapeutic with small size YCW NPs and anti-PD-L1 for B16. (**A-B**) Quantification of bioluminescence signal of primary tumors (**A**) and distant tumors (**B**) observed on day 8, 11, 14, 17 (UnTx: n = 4; aPD-L1: n = 3; Small NPs: n = 4; Combine: n = 4). (**C**) Weight curves of mice in different groups during treatment (n = 4). (**D**) Imaging of mice in four groups on day 14 and day 17. Statistical significance was obtained by Student's t tests (two-tailed). *** $P < 0.005$; * $P < 0.05$. Data are means ± SD.

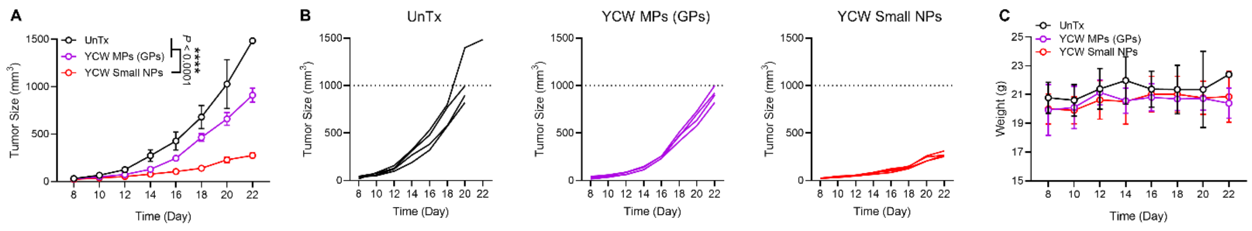

Supplementary Fig. 17. Treatment efficiency of YCW small NPs and YCW MPs (GPs). (**A**) Average and (**B**) individual tumor growth curves in different treatment group, including UnTx, YCW MPs (GPs), YCW small NPs (n = 4). (**C**) Weight of mice in three groups during treatment (n = 4). Statistical significance was obtained by one-way ANOVA using the Tukey post-test. **** $P < 0.0001$. Data are means ± SD.
