# Supplementary material for: Yeast-derived nanoparticles remodel the immunosuppressive microenvironment in tumor and tumor-draining lymph nodes to suppress tumor growth
Source: Nat Commun. 2022 Jan 10;13:110. doi: 10.1038/s41467-021-27750-2 (PMC8748771; doi:10.1038/s41467-021-27750-2)
Supplement: Supplementary file 2 — Reporting Summary [file 41467_2021_27750_MOESM2_ESM.pdf]

nature research

Corresponding author(s): Chao Wang, Jingrun Chen, Jianhong Chu

Last updated by author(s): Nov 29, 2021

# Reporting Summary

Nature Research wishes to improve the reproducibility of the work that we publish. This form provides structure for consistency and transparency in reporting. For further information on Nature Research policies, see our Editorial Policies and the Editorial Policy Checklist.

## Statistics

For all statistical analyses, confirm that the following items are present in the figure legend, table legend, main text, or Methods section.

| n/a | Confirmed | |
|---|---|---|
| ☐ | ☒ | The exact sample size (*n*) for each experimental group/condition, given as a discrete number and unit of measurement |
| ☐ | ☒ | A statement on whether measurements were taken from distinct samples or whether the same sample was measured repeatedly |
| ☐ | ☒ | The statistical test(s) used AND whether they are one- or two-sided<br>*Only common tests should be described solely by name; describe more complex techniques in the Methods section.* |
| ☐ | ☒ | A description of all covariates tested |
| ☐ | ☒ | A description of any assumptions or corrections, such as tests of normality and adjustment for multiple comparisons |
| ☐ | ☒ | A full description of the statistical parameters including central tendency (e.g. means) or other basic estimates (e.g. regression coefficient) AND variation (e.g. standard deviation) or associated estimates of uncertainty (e.g. confidence intervals) |
| ☐ | ☒ | For null hypothesis testing, the test statistic (e.g. *F*, *t*, *r*) with confidence intervals, effect sizes, degrees of freedom and *P* value noted<br>*Give P values as exact values whenever suitable.* |
| ☒ | ☐ | For Bayesian analysis, information on the choice of priors and Markov chain Monte Carlo settings |
| ☒ | ☐ | For hierarchical and complex designs, identification of the appropriate level for tests and full reporting of outcomes |
| ☒ | ☐ | Estimates of effect sizes (e.g. Cohen's *d*, Pearson's *r*), indicating how they were calculated |

*Our web collection on statistics for biologists contains articles on many of the points above.*

## Software and code

Policy information about availability of computer code

| | |
|---|---|
| Data collection | No software was used for date collection |
| Data analysis | All statistical analysis were performed on Graphpad Prism 8. Living Image software (Perkin Elmer) was used to analyze bioluminescent images. All the flow cytometry data were processed using FlowJo (version 10.4., Becton, Dickinson & Company). Western blotting data were analyzed by ImageJ (version 6). |

For manuscripts utilizing custom algorithms or software that are central to the research but not yet described in published literature, software must be made available to editors and reviewers. We strongly encourage code deposition in a community repository (e.g. GitHub). See the Nature Research guidelines for submitting code & software for further information.

## Data

Policy information about availability of data

All manuscripts must include a data availability statement. This statement should provide the following information, where applicable:

- Accession codes, unique identifiers, or web links for publicly available datasets
- A list of figures that have associated raw data
- A description of any restrictions on data availability

The raw data can be found in the Source Data. Source data are provided with this paper. The authors declare that all other data supporting the findings of this study are within the article and its Supplementary Information file. The protein database has been provided in Source Data.

# Field-specific reporting

Please select the one below that is the best fit for your research. If you are not sure, read the appropriate sections before making your selection.

☒ Life sciences ☐ Behavioural & social sciences ☐ Ecological, evolutionary & environmental sciences

For a reference copy of the document with all sections, see nature.com/documents/nr-reporting-summary-flat.pdf

# Life sciences study design

All studies must disclose on these points even when the disclosure is negative.

| | |
|---|---|
| Sample size | No statistical methods were used to predetermine the sample sizes. It is impossible to predict the magnitude of experimental variation between animals based on our current knowledge. The group sizes (at least three animals per treatment group) represents the minimum number animals needed to reach statistical significance ($p < 0.05$) between experimental groups. |
| Data exclusions | No data were excluded. |
| Replication | Experiments were repeated and experimental findings were reproducible. All experiments were run at least in triplicate. |
| Randomization | All experimental samples or models were allocated randomly to each group. |
| Blinding | No blinding was done in this study. Most of the studies contained multiple steps (including the material preparation, tumor therapeutics, and so on) and the scientists must keep careful track of every condition. It would be exceedingly difficult to blind such studies. |

# Behavioural & social sciences study design

All studies must disclose on these points even when the disclosure is negative.

| | |
|---|---|
| Study description | *Briefly describe the study type including whether data are quantitative, qualitative, or mixed-methods (e.g. qualitative cross-sectional, quantitative experimental, mixed-methods case study).* |
| Research sample | *State the research sample (e.g. Harvard university undergraduates, villagers in rural India) and provide relevant demographic information (e.g. age, sex) and indicate whether the sample is representative. Provide a rationale for the study sample chosen. For studies involving existing datasets, please describe the dataset and source.* |
| Sampling strategy | *Describe the sampling procedure (e.g. random, snowball, stratified, convenience). Describe the statistical methods that were used to predetermine sample size OR if no sample-size calculation was performed, describe how sample sizes were chosen and provide a rationale for why these sample sizes are sufficient. For qualitative data, please indicate whether data saturation was considered, and what criteria were used to decide that no further sampling was needed.* |
| Data collection | *Provide details about the data collection procedure, including the instruments or devices used to record the data (e.g. pen and paper, computer, eye tracker, video or audio equipment) whether anyone was present besides the participant(s) and the researcher, and whether the researcher was blind to experimental condition and/or the study hypothesis during data collection.* |
| Timing | *Indicate the start and stop dates of data collection. If there is a gap between collection periods, state the dates for each sample cohort.* |
| Data exclusions | *If no data were excluded from the analyses, state so OR if data were excluded, provide the exact number of exclusions and the rationale behind them, indicating whether exclusion criteria were pre-established.* |
| Non-participation | *State how many participants dropped out/declined participation and the reason(s) given OR provide response rate OR state that no participants dropped out/declined participation.* |
| Randomization | *If participants were not allocated into experimental groups, state so OR describe how participants were allocated to groups, and if allocation was not random, describe how covariates were controlled.* |

# Ecological, evolutionary & environmental sciences study design

All studies must disclose on these points even when the disclosure is negative.

| | |
|---|---|
| Study description | *Briefly describe the study. For quantitative data include treatment factors and interactions, design structure (e.g. factorial, nested, hierarchical), nature and number of experimental units and replicates.* |
| Research sample | *Describe the research sample (e.g. a group of tagged Passer domesticus, all Stenocereus thurberi within Organ Pipe Cactus National Monument), and provide a rationale for the sample choice. When relevant, describe the organism taxa, source, sex, age range and* |

| | |
|---|---|
| | *any manipulations. State what population the sample is meant to represent when applicable. For studies involving existing datasets, describe the data and its source.* |
| Sampling strategy | *Note the sampling procedure. Describe the statistical methods that were used to predetermine sample size OR if no sample-size calculation was performed, describe how sample sizes were chosen and provide a rationale for why these sample sizes are sufficient.* |
| Data collection | *Describe the data collection procedure, including who recorded the data and how.* |
| Timing and spatial scale | *Indicate the start and stop dates of data collection, noting the frequency and periodicity of sampling and providing a rationale for these choices. If there is a gap between collection periods, state the dates for each sample cohort. Specify the spatial scale from which the data are taken* |
| Data exclusions | *If no data were excluded from the analyses, state so OR if data were excluded, describe the exclusions and the rationale behind them, indicating whether exclusion criteria were pre-established.* |
| Reproducibility | *Describe the measures taken to verify the reproducibility of experimental findings. For each experiment, note whether any attempts to repeat the experiment failed OR state that all attempts to repeat the experiment were successful.* |
| Randomization | *Describe how samples/organisms/participants were allocated into groups. If allocation was not random, describe how covariates were controlled. If this is not relevant to your study, explain why.* |
| Blinding | *Describe the extent of blinding used during data acquisition and analysis. If blinding was not possible, describe why OR explain why blinding was not relevant to your study.* |

Did the study involve field work? ☐ Yes ☐ No

## Field work, collection and transport

| | |
|---|---|
| Field conditions | *Describe the study conditions for field work, providing relevant parameters (e.g. temperature, rainfall).* |
| Location | *State the location of the sampling or experiment, providing relevant parameters (e.g. latitude and longitude, elevation, water depth).* |
| Access & import/export | *Describe the efforts you have made to access habitats and to collect and import/export your samples in a responsible manner and in compliance with local, national and international laws, noting any permits that were obtained (give the name of the issuing authority, the date of issue, and any identifying information).* |
| Disturbance | *Describe any disturbance caused by the study and how it was minimized.* |

# Reporting for specific materials, systems and methods

We require information from authors about some types of materials, experimental systems and methods used in many studies. Here, indicate whether each material, system or method listed is relevant to your study. If you are not sure if a list item applies to your research, read the appropriate section before selecting a response.

### Materials & experimental systems

| n/a | Involved in the study | |
|---|---|---|
| ☐ | ☒ | Antibodies |
| ☐ | ☒ | Eukaryotic cell lines |
| ☒ | ☐ | Palaeontology and archaeology |
| ☐ | ☒ | Animals and other organisms |
| ☒ | ☐ | Human research participants |
| ☒ | ☐ | Clinical data |
| ☒ | ☐ | Dual use research of concern |

### Methods

| n/a | Involved in the study | |
|---|---|---|
| ☒ | ☐ | ChIP-seq |
| ☐ | ☒ | Flow cytometry |
| ☒ | ☐ | MRI-based neuroimaging |

## Antibodies

Antibodies used

1. anti-CD80-PE (Biolegend, clone: 16-10A1, Catalog: 104708),
2. anti-CD86-APC (Biolegend, clone: GL-1, Catalog: 105012),
3. anti-CD3-FITC (Biolegend, clone: 17A2, Catalog: 100204),
4. anti-CD4-PE (Biolegend, clone: GK1.5, Catalog: 100408),
5. anti-CD8a-APC (Biolegend, clone: 53-6.7, Catalog: 100712),
6. anti-PD-1-APC (Biolegend, clone: 29F.1A12, Catalog: 135210),
7. anti-CD11b-FITC (Biolegend, clone: M1/70, Catalog: 101206),
8. anti-Gr-1-APC (Biolegend, clone: RB6-8C5, Catalog: 108412),
9. anti-CD4-APC (Biolegend, clone: GK1.5, Catalog: 100412),
10. anti-FOXP3-PE (Biolegend, clone: MF-14, Catalog: 126403),
11. anti-F4/80-FITC (Biolegend, clone: BM8, Catalog: 123108),

12. anti-CD206-APC (Biolegend, clone: C068C2, Catalog: 141706),
13. anti-CD86-PE (Biolegend, clone: GL-1, Catalog: 105008),
14. anti-CD80-APC (Biolegend, clone: 16-10A1, Catalog: 104714),
15. anti-CD69-APC (Biolegend, clone: H1.2F3, Catalog: 104514),
16. anti-CD40-APC (Biolegend, clone: 3/23, Catalog: 124612),
17. anti-I-A/I-E-PE (Biolegend, clone: M5/114.15.2, Catalog: 107608),
18. anti-PD-L1-PE (Biolegend, clone: 10F.9G2, Catalog: 124308),
19. anti-CD11c-FITC (Biolegend, clone:N418, Catalog: 117306),
20. anti-CD45-FITC (Biolegend, clone: 30-F11, Catalog: 103108),
21. anti-Percp-CD11b (Biolegend, clone: M1/70, Catalog: 101230),
22. anti-p-P65 (Abclonal, Rabbit, Catalog: AP0475),
23. anti-GAPDH (Servicebio, Rabbit, Catalog: GB11002),
24. anti-TLR-2 (Abcam, Rabbit, Catalog: ab209216),
25. anti-p-Syk (Cell Signaling Technology, Rabbit, Catalog: #2710),
26. anti-MyD88 (Abcam, Rabbit, Catalog: ab219413),
27. anti-Dectin-1 (Absin, Rabbit, Catalog: abs124190),
28. Goat anti-Rabbit IgG (H+L)-HRP (Ray Antibody, Catalog: RM3002).

Validation

All antibodies were verified by the supplier and each lot has been qualitically tested. All validation statements can be found on the corresponding antibody websites as below:
1. PE anti-mouse CD80 Antibody: https://www.biolegend.com/en-us/products/pe-anti-mouse-cd80-antibody-43;
2. APC anti-mouse CD86 Antibody: https://www.biolegend.com/en-us/products/apc-anti-mouse-cd86-antibody-2896;
3. FITC anti-mouse CD3 Antibody: https://www.biolegend.com/en-us/products/fitc-anti-mouse-cd3-antibody-45;
4. PE anti-mouse CD4 Antibody: https://www.biolegend.com/en-us/products/pe-anti-mouse-cd4-antibody-250;
5. APC anti-mouse CD8a Antibody: https://www.biolegend.com/en-us/products/apc-anti-mouse-cd8a-antibody-150;
6. APC anti-mouse CD279 (PD-1) Antibody: https://www.biolegend.com/en-us/products/apc-anti-mouse-cd279-pd-1-antibody-6497;
7. FITC anti-mouse/human CD11b Antibody: https://www.biolegend.com/en-us/products/fitc-anti-mouse-human-cd11b-antibody-347;
8. APC anti-mouse Ly-6G/Ly-6C (Gr-1) Antibody: https://www.biolegend.com/en-us/products/apc-anti-mouse-ly-6g-ly-6c-gr-1-antibody-456;
9. APC anti-mouse CD4 Antibody: https://www.biolegend.com/en-us/products/apc-anti-mouse-cd4-antibody-245;
10. PE anti-mouse FOXP3 Antibody: https://www.biolegend.com/en-us/products/pe-anti-mouse-foxp3-antibody-4660;
11. FITC anti-mouse F4/80 Antibody: https://www.biolegend.com/en-us/products/fitc-anti-mouse-f4-80-antibody-4067;
12. APC anti-mouse CD206 (MMR) Antibody: https://www.biolegend.com/en-us/products/apc-anti-mouse-cd206-mmr-antibody-7425;
13. PE anti-mouse CD86 Antibody: https://www.biolegend.com/en-us/products/pe-anti-mouse-cd86-antibody-256;
14. APC anti-mouse CD80 Antibody: https://www.biolegend.com/en-us/products/apc-anti-mouse-cd80-antibody-2340;
15. APC anti-mouse CD69 Antibody: https://www.biolegend.com/en-us/products/apc-anti-mouse-cd69-antibody-3169;
16. APC anti-mouse CD40 Antibody: https://www.biolegend.com/en-us/products/apc-anti-mouse-cd40-antibody-4984;
17. PE anti-mouse I-A/I-E Antibody: https://www.biolegend.com/en-us/products/pe-anti-mouse-i-a-i-e-antibody-367;
18. PE anti-mouse CD274 (B7-H1, PD-L1) Antibody: https://www.biolegend.com/en-us/products/pe-anti-mouse-cd274-b7-h1-pd-l1-antibody-4497;
19. FITC anti-mouse CD11c Antibody: https://www.biolegend.com/en-us/products/fitc-anti-mouse-cd11c-antibody-1815;
20. FITC anti-mouse CD45 Antibody: https://www.biolegend.com/en-us/products/fitc-anti-mouse-cd45-antibody-99;
21. PerCP anti-mouse/human CD11b Antibody: https://www.biolegend.com/en-us/products/percp-anti-mouse-human-cd11b-antibody-4315;
22. anti-p-Syk: https://www.cellsignal.cn/products/primary-antibodies/phospho-syk-tyr525-526-c87c1-rabbit-mab/2710?_=1638110111169&Ntt=phospho-Syk&tahead=true;
23. anti-p-P65: https://abclonal.com.cn/search/index?catid=56&keyword=Anti-p-P65&__hash__=6666cd76f96956469e7be39d750cc7d9_99d02f472a22c6725a7323c52965b8c6&Searchbar=;
24. anti-GAPDH: https://www.servicebio.cn/promotionTrialDetail?id=7252;
25. anti-TLR-2: https://www.abcam.cn/tlr2-antibody-epr20303-ab209217.html;
26. anti-MyD88: https://www.abcam.cn/myd88-antibody-epr21824-ab219413.html;
27. anti-Dectin-1: https://www.absin.cn/rabbit-anti-beta-glucan-receptor-polyclonal-antibody/abs124190.html.

# Eukaryotic cell lines

Policy information about cell lines

Cell line source(s)

Murine CT26 colon cancer cells, murine B16 melanoma cells, RAW264.7 and DC2.4 cells were all obtained from the Cell Bank, Shanghai Institutes for Biological Sciences, Chinese Academy of Sciences.

Authentication

Authentication of cells was not performed after receipt.

Mycoplasma contamination

All cell lines were tested for mycoplasma contamination. No mycoplasma contamination was found.

Commonly misidentified lines
(See ICLAC register)

No commonly misidentified cell lines were used in this study.

## Palaeontology and Archaeology

| | |
|---|---|
| Specimen provenance | *Provide provenance information for specimens and describe permits that were obtained for the work (including the name of the issuing authority, the date of issue, and any identifying information).* |
| Specimen deposition | *Indicate where the specimens have been deposited to permit free access by other researchers.* |
| Dating methods | *If new dates are provided, describe how they were obtained (e.g. collection, storage, sample pretreatment and measurement), where they were obtained (i.e. lab name), the calibration program and the protocol for quality assurance OR state that no new dates are provided.* |

☐ Tick this box to confirm that the raw and calibrated dates are available in the paper or in Supplementary Information.

| | |
|---|---|
| Ethics oversight | *Identify the organization(s) that approved or provided guidance on the study protocol, OR state that no ethical approval or guidance was required and explain why not.* |

Note that full information on the approval of the study protocol must also be provided in the manuscript.

## Animals and other organisms

Policy information about studies involving animals; ARRIVE guidelines recommended for reporting animal research

| | |
|---|---|
| Laboratory animals | 6-8-week-old Balb/c female mice (18 ± 2g) and 6-8-week-old C57BL/6 female mice (18± 2g) were purchased from Laboratory Animal Center of Soochow University. Mice were housed in groups of 6 mice per individually ventilated cage in a 12-h light-dark cycle (8:00-20:00 light; 20:00-8:00 dark), with constant room temperature (21± 1°C) and relative humidity (40-70%). All mice had access to food ad water ad libitum. |
| Wild animals | The study did not involve wild animals. |
| Field-collected samples | The study did not involve samples collected from the field. |
| Ethics oversight | The experimental protocols were approved by the Institutional Animal Care and Use Committee (IACUC) of the Animal Experiment Center of Soochow University (Suzhou, China, Approval number: SUDA20210201A02). All animal experimental procedures were performed in accordance with the Regulations for the Administration of Affairs Concerning Experimental Animals approved by the State Council of the People’s Republic of China. |

Note that full information on the approval of the study protocol must also be provided in the manuscript.

## Human research participants

Policy information about studies involving human research participants

| | |
|---|---|
| Population characteristics | *Describe the covariate-relevant population characteristics of the human research participants (e.g. age, gender, genotypic information, past and current diagnosis and treatment categories). If you filled out the behavioural & social sciences study design questions and have nothing to add here, write "See above."* |
| Recruitment | *Describe how participants were recruited. Outline any potential self-selection bias or other biases that may be present and how these are likely to impact results.* |
| Ethics oversight | *Identify the organization(s) that approved the study protocol.* |

Note that full information on the approval of the study protocol must also be provided in the manuscript.

## Clinical data

Policy information about clinical studies

All manuscripts should comply with the ICMJEguidelines for publication of clinical research and a completedCONSORT checklist must be included with all submissions.

| | |
|---|---|
| Clinical trial registration | *Provide the trial registration number from ClinicalTrials.gov or an equivalent agency.* |
| Study protocol | *Note where the full trial protocol can be accessed OR if not available, explain why.* |
| Data collection | *Describe the settings and locales of data collection, noting the time periods of recruitment and data collection.* |
| Outcomes | *Describe how you pre-defined primary and secondary outcome measures and how you assessed these measures.* |

## Dual use research of concern

Policy information about dual use research of concern

### Hazards

Could the accidental, deliberate or reckless misuse of agents or technologies generated in the work, or the application of information presented in the manuscript, pose a threat to:

| No | Yes | |
|---|---|---|
| ☐ | ☐ | Public health |
| ☐ | ☐ | National security |
| ☐ | ☐ | Crops and/or livestock |
| ☐ | ☐ | Ecosystems |
| ☐ | ☐ | Any other significant area |

## Experiments of concern

Does the work involve any of these experiments of concern:

| No | Yes | |
|---|---|---|
| ☐ | ☐ | Demonstrate how to render a vaccine ineffective |
| ☐ | ☐ | Confer resistance to therapeutically useful antibiotics or antiviral agents |
| ☐ | ☐ | Enhance the virulence of a pathogen or render a nonpathogen virulent |
| ☐ | ☐ | Increase transmissibility of a pathogen |
| ☐ | ☐ | Alter the host range of a pathogen |
| ☐ | ☐ | Enable evasion of diagnostic/detection modalities |
| ☐ | ☐ | Enable the weaponization of a biological agent or toxin |
| ☐ | ☐ | Any other potentially harmful combination of experiments and agents |

# ChIP-seq

## Data deposition

☐ Confirm that both raw and final processed data have been deposited in a public database such as GEO.

☐ Confirm that you have deposited or provided access to graph files (e.g. BED files) for the called peaks.

| | |
|---|---|
| Data access links<br>*May remain private before publication.* | *For "Initial submission" or "Revised version" documents, provide reviewer access links. For your "Final submission" document, provide a link to the deposited data.* |
| Files in database submission | *Provide a list of all files available in the database submission.* |
| Genome browser session<br>(e.g. UCSC) | *Provide a link to an anonymized genome browser session for "Initial submission" and "Revised version" documents only, to enable peer review. Write "no longer applicable" for "Final submission" documents.* |

## Methodology

| | |
|---|---|
| Replicates | *Describe the experimental replicates, specifying number, type and replicate agreement.* |
| Sequencing depth | *Describe the sequencing depth for each experiment, providing the total number of reads, uniquely mapped reads, length of reads and whether they were paired- or single-end.* |
| Antibodies | *Describe the antibodies used for the ChIP-seq experiments; as applicable, provide supplier name, catalog number, clone name, and lot number.* |
| Peak calling parameters | *Specify the command line program and parameters used for read mapping and peak calling, including the ChIP, control and index files used.* |
| Data quality | *Describe the methods used to ensure data quality in full detail, including how many peaks are at FDR 5% and above 5-fold enrichment.* |
| Software | *Describe the software used to collect and analyze the ChIP-seq data. For custom code that has been deposited into a community repository, provide accession details.* |

# Flow Cytometry

## Plots

Confirm that:

☒ The axis labels state the marker and fluorochrome used (e.g. CD4-FITC).

☒ The axis scales are clearly visible. Include numbers along axes only for bottom left plot of group (a 'group' is an analysis of identical markers).

☒ All plots are contour plots with outliers or pseudocolor plots.

☒ A numerical value for number of cells or percentage (with statistics) is provided.

## Methodology

| | |
|---|---|
| Sample preparation | Tumor and tumor draining lymph nodes were harvested from mice. The tissue was cut into small pieces before being homogenized in buffers to form single cell suspensions in the presence of digestive enzymes. For all samples, cells were firstly stained with antibodies against surface antigens. In some experiments, cells were subsequently fixed, permeablized and stained for intracellular antigens. |
| Instrument | BD AccuritTM C6 Plus |
| Software | FlowJo X 10.0.7r2 |
| Cell population abundance | No sorting was performed |
| Gating strategy | Gating strategies are referred to those described in the website (https://www.bio-rad-antibodies.com/blog/a-guide-to-gating-in-flow-cytometry.html). In general, cells were first gated on FSC/SSC. Surface and intracellular antigen gating was performed on the first gating cell population. The cell populations within the gate were further analysised based on expression of markers. Single positive straining were uesd to determing the "true" positive. Gating was then based on positive level (after proper compensation). The detailed gating strategy could be found in supporting information. |

☒ Tick this box to confirm that a figure exemplifying the gating strategy is provided in the Supplementary Information.

# Magnetic resonance imaging

## Experimental design

| | |
|---|---|
| Design type | *Indicate task or resting state; event-related or block design.* |
| Design specifications | *Specify the number of blocks, trials or experimental units per session and/or subject, and specify the length of each trial or block (if trials are blocked) and interval between trials.* |
| Behavioral performance measures | *State number and/or type of variables recorded (e.g. correct button press, response time) and what statistics were used to establish that the subjects were performing the task as expected (e.g. mean, range, and/or standard deviation across subjects).* |

## Acquisition

| | |
|---|---|
| Imaging type(s) | *Specify: functional, structural, diffusion, perfusion.* |
| Field strength | *Specify in Tesla* |
| Sequence & imaging parameters | *Specify the pulse sequence type (gradient echo, spin echo, etc.), imaging type (EPI, spiral, etc.), field of view, matrix size, slice thickness, orientation and TE/TR/flip angle.* |
| Area of acquisition | *State whether a whole brain scan was used OR define the area of acquisition, describing how the region was determined.* |

Diffusion MRI ☐ Used ☐ Not used

## Preprocessing

| | |
|---|---|
| Preprocessing software | *Provide detail on software version and revision number and on specific parameters (model/functions, brain extraction, segmentation, smoothing kernel size, etc.).* |
| Normalization | *If data were normalized/standardized, describe the approach(es): specify linear or non-linear and define image types used for transformation OR indicate that data were not normalized and explain rationale for lack of normalization.* |
| Normalization template | *Describe the template used for normalization/transformation, specifying subject space or group standardized space (e.g. original Talairach, MNI305, ICBM152) OR indicate that the data were not normalized.* |

| | |
|---|---|
| Noise and artifact removal | *Describe your procedure(s) for artifact and structured noise removal, specifying motion parameters, tissue signals and physiological signals (heart rate, respiration).* |
| Volume censoring | *Define your software and/or method and criteria for volume censoring, and state the extent of such censoring.* |

## Statistical modeling & inference

| | |
|---|---|
| Model type and settings | *Specify type (mass univariate, multivariate, RSA, predictive, etc.) and describe essential details of the model at the first and second levels (e.g. fixed, random or mixed effects; drift or auto-correlation).* |
| Effect(s) tested | *Define precise effect in terms of the task or stimulus conditions instead of psychological concepts and indicate whether ANOVA or factorial designs were used.* |

Specify type of analysis: ☐ Whole brain ☐ ROI-based ☐ Both

| | |
|---|---|
| Statistic type for inference (See Eklund et al. 2016) | *Specify voxel-wise or cluster-wise and report all relevant parameters for cluster-wise methods.* |
| Correction | *Describe the type of correction and how it is obtained for multiple comparisons (e.g. FWE, FDR, permutation or Monte Carlo).* |

## Models & analysis

| n/a | Involved in the study |
|---|---|
| ☐ | ☐ Functional and/or effective connectivity |
| ☐ | ☐ Graph analysis |
| ☐ | ☐ Multivariate modeling or predictive analysis |

| | |
|---|---|
| Functional and/or effective connectivity | *Report the measures of dependence used and the model details (e.g. Pearson correlation, partial correlation, mutual information).* |
| Graph analysis | *Report the dependent variable and connectivity measure, specifying weighted graph or binarized graph, subject- or group-level, and the global and/or node summaries used (e.g. clustering coefficient, efficiency, etc.).* |
| Multivariate modeling and predictive analysis | *Specify independent variables, features extraction and dimension reduction, model, training and evaluation metrics.* |
